# “They always disfavor me!”: Parental conditional regard undermines teenage sibling relationships through raising competition and perceived disfavoritism

**DOI:** 10.1111/jora.70071

**Published:** 2025-09-08

**Authors:** Sarah Teresa Steffgen, Nantje Otterpohl, Avi Assor, Yaniv Kanat‐Maymon, Bat El Gueta, Malte Schwinger

**Affiliations:** ^1^ Department of Educational Psychology Philipps‐University Marburg Marburg Germany; ^2^ Educational Psychology Program, School of Education Ben Gurion University of the Negev Beer‐Sheva Israel; ^3^ School of Psychology Reichman University Herzliya Israel

**Keywords:** competition, favoritism, parental conditional regard, sibling conflict, sibling relationship

## Abstract

Conditional regard defined as adolescents' perception that parents' affection hinges upon whether they meet parental expectations affects their skills to build stable and sustainable relationships. However, its role in sibling relationships remains unexplored. This study investigates how conditional regard relates to sibling competition, favoritism, and conflict in 201 teenage sibling dyads. Actor‐Partner Interdependence Mediation Models showed that conditional regard was associated with lower sibling relationship quality. Similarities between siblings' reports were low, and siblings' perceptions of parenting mainly predicted their own perceptions of sibling relationships. Competition and feeling disfavored mediated the negative effects of conditional regard on conflict. Feeling favored was predicted by conditional positive regard but did not mediate effects. Our findings highlight the potential of conditional regard to undermine sibling bonds.

## INTRODUCTION

Parental conditional regard refers to a parenting behavior where parents' appreciation of their child varies depending on whether the child lives up to parental expectations (Assor et al., 2004). Self‐determination theory (SDT; Deci & Ryan, 2000; Ryan & Deci, 2017) posits that this type of parenting frustrates children's basic psychological needs, making it a psychologically controlling parenting style. Previous research has consistently shown that parental conditional regard frustrates basic psychological needs (Haines & Schutte, 2022) and brings negative effects on various developmental outcomes of children, such as the development of contingent self‐esteem, introjected motivation, and suppressive and dysregulated emotion regulation (Assor et al., 2004, 2014; Haines & Schutte, 2022; Roth et al., 2009; Roth & Assor, 2012). Additionally, conditional regard affects key skills that are important for building high‐quality relationships, such as the capacity to disclose negative emotions (Roth & Assor, 2012) or providing need support to another person (Kanat‐Maymon et al., 2016; Moller et al., 2019). Therefore, parental conditional regard affects children's current and future relationships with their parents, peers, and romantic partners (Kanat‐Maymon et al., 2016; Moller et al., 2019; Roth et al., 2009). Surprisingly, no research has investigated how it shapes one of an individual's longest relationships—the relationship with siblings. Conditional regard signals that parental love and attention are sources of need satisfaction that one has to earn and possibly compete for with others who fight for the same, such as siblings. Founded on SDT, the purpose of the present study was to conduct an initial investigation into the effects of parental conditional regard on different facets of the sibling relationship.

### Conditional regard and its harmful impact on sibling relationships

Conditional regard is examined in two distinct forms: the increase of affection when the child succeeds and meets parental expectations (parental conditional positive regard, PCPR), and the decrease of affection when the child fails to do so (parental conditional negative regard, PCNR; Roth et al., 2009). From a SDT perspective, both forms of conditional regard are psychologically controlling because they use the fulfillment of children's basic psychological need for relatedness, that is, the need to feel cared for and to belong, as a reinforcer (Ryan & Deci, 2017). This leads to children striving to meet their parents' expectations to gain parental approval, which undermines their need for autonomy (i.e., the need for psychological freedom and freedom in their own actions). Consequently, conditional regard leads to autonomy frustration and a diluted relatedness satisfaction (Haines & Schutte, 2022; Kanat‐Maymon et al., 2016, 2023; Moller et al., 2019; Steffgen et al., 2022). This need frustration undermines the parent–child relationship, for example, by fostering resentment (Assor et al., 2004; Roth et al., 2009). Moreover, conditional regard also reduces relationship quality and attachment security in other close relationships, such as those with romantic partners and best friends (Kanat‐Maymon et al., 2016; Moller et al., 2019). Impaired emotion regulation, reduced capacity to disclose negative emotions, and decreased ability to support others in their negative emotions have been identified as key mechanisms underlying this effect (Roth & Assor, 2012).

Despite the negative effects of conditional regard on children's close relationships, its impact on sibling relationships remains unexplored. Sibling relationships are unique due to their more egalitarian nature, which is distinct from the hierarchical parent–child relationship (Cicirelli, 1995). Furthermore, sibling relationships are also distinct from relationships with romantic partners or friends because of their lifelong, biologically connected bond that involves shared environments and unique family expectations (Cicirelli, 1995). Sibling relationships are emotionally intense and contribute significantly to individuals' need satisfaction, psychological adjustment, and well‐being throughout life (Barberis et al., 2022; Gilligan et al., 2020; Waldinger, 2007).

Research suggests that parental conditional regard may impair sibling relationship quality, particularly by promoting sibling conflict, an important component of sibling relationship quality characterized by fighting or insulting one another (Jensen, Killoren, et al., 2023). Maladaptive parenting has been meta‐analytically linked to sibling conflicts (Liu & Rahman, 2022). Moreover, parental psychological control, a concept closely related to PCNR, has been linked to relational aggression between siblings (Campione‐Barr et al., 2014) as well as psychological control between siblings mediated by siblings' need frustration (van der Kaap‐Deeder, 2021). Thus, the frustration of basic psychological needs is a crucial mechanism through which conditional regard may affect sibling relationship quality, alongside other factors such as social learning and emotional development. Research has also demonstrated that the frustration of basic psychological needs is associated with negative interpersonal behavior, such as aggression (Kuzucu & Şimşek, 2013) and externalizing behavior (Vandenkerckhove et al., 2019). Furthermore, need frustration in romantic partners has been shown to predict their initiation of relationship conflicts (Vanhee et al., 2018). When parents are psychologically controlling in managing sibling conflicts, children experience lower sibling relationship quality (van der Kaap‐Deeder, 2021). The existing findings suggest that parental conditional regard may also diminish sibling relationship quality by eliciting conflicts in the first place.

### Parental conditional regard and sibling dynamics

Parental conditional regard creates diluted and frustrated needs, leading children to have a heightened focus on seeking and striving for parental approval (Kanat‐Maymon et al., 2023). In the context of sibling relationships, children share parental approval, attention, or recognition with their siblings, making them potential rivals for these limited parental resources. We assume that the heightened focus on striving for parental approval has potential consequences for sibling dynamics that link parental conditional regard with impaired sibling relationship quality.

One potential consequence is competition between siblings. Conditional regard fosters a reliance on external validation from parents and an orientation toward external standards of success and achievement, rather than developing a sense of one's true values. Consequently, children become more focused on grades and on demonstrating and showcasing their own competence to others (Roth et al., 2009; Steffgen et al., 2022). In the context of sibling relationships, this mindset may lead to a competitive dynamic, where siblings feel pressure to outperform each other in domains where they receive conditional regard, such as academics, in order to maintain parental approval and fulfill their basic psychological needs.

Another potential consequence is that siblings compare the affection they receive with that given to their sibling, resulting in perceived parental differential affection, a type of parental differential treatment (Daniels & Plomin, 1985). Moreover, children may feel that one sibling is favored over the other. Parental favoritism involves that siblings perceive parental differential treatment as unfair, in contrast to appropriate due to the different needs of siblings (Finzi‐Dottan & Cohen, 2011), and is a crucial component in determining the negative impact of parental differential treatment (McHale et al., 2000). We propose that parental conditional regard promotes a sense of neediness in children, which can lead to the perception of being short‐changed and *disfavored*, regardless of whether they objectively receive more or less affection compared to their siblings. This may particularly be pronounced with PCNR due to its failure‐oriented nature. In contrast to PCNR, which is associated with feelings of self‐devaluation, PCPR is success‐oriented and accompanied by feelings of self‐aggrandizement (Assor & Tal, 2012), which may even promote feelings of being *favored*, that is, receiving more affection in comparison to siblings.

Both competition and disfavoritism have been found to diminish sibling relationship quality and promote sibling conflicts in particular. Research shows that a comparison orientation is linked to sibling conflict (Jensen et al., 2015), suggesting that constant competition for parental approval and attention as well as the comparison of perceived affection given to oneself and one's sibling likely contribute to feelings of resentment among siblings. Further evidence for these relations comes from research linking competition to fights and insults between siblings (Bojanowski et al., 2015) and hostility toward siblings (Meunier et al., 2012), as well as more externalizing problems (Richmond et al., 2005). In contrast, feeling slightly favored was perceived as fair (Gozu & Newman, 2020), and might therefore not diminish sibling relationship quality. Therefore, we assume that sibling competition and feelings of being disfavored or favored are important outcomes of parental conditional regard, and that competition and feeling disfavored, but not feeling favored, depict potential mechanisms of how parental conditional regard promotes sibling conflicts.

### Differential perceptions and interdependence between siblings

While the negative effects of conditional regard have primarily been studied for the individual child (e.g., examining motivational and emotional development), exploring its potential impact on the sibling relationship adds complexity. Within the same family, two children are likely exposed to the same detrimental parenting style (shared environment), potentially leading to cumulative effects in the sibling relationship. Yet, children do not necessarily experience the same degree of parental conditional regard. Research suggests only slight agreement between siblings in their perceptions of parenting (e.g., for autonomy support, *r* = .30; van der Kaap‐Deeder et al., 2015). Whether children interpret the same parenting style differently or actually receive different levels of conditional regard (nonshared environment), these varying perceptions can differentially affect how siblings contribute to and perceive their sibling relationship. Additionally, birth order effects are prevalent for various types of sibling relationship facets. For instance, younger siblings are more oriented toward comparison (Jensen et al., 2015) and receive more affection (Jensen & McHale, 2017). Thus, it is important to examine whether parental conditional regard affects older and younger siblings differently. For example, due to their higher comparison orientation, younger siblings may be more prone to developing stronger competition and perceiving favoritism in conditionally regarding settings. On the other hand, favoritism may be more pronounced when PCR is perceived, with older siblings being more affected as they receive less affection overall. Considering the potentially divergent perceptions of parental conditional regard between siblings and the potentially distinguishable effects between older and younger siblings provides a more comprehensive understanding of how conditional regard influences sibling dynamics.

### Hypotheses

This study aimed for an initial investigation of the effects of parental conditional regard on sibling relationships. We hypothesized that PCNR and PCPR are associated with higher sibling conflicts. We expected this relationship to be mediated by sibling competition and favoritism, particularly feeling disfavored. More specifically, we hypothesized that PCNR and PCPR promote an external norm of reference and a competitive mindset, which would result in higher competition between siblings. Further, we expected that parental conditional regard would promote perceived favoritism. Consistent with the failure and success orientation in PCNR and PCPR, we expected that children would have a higher tendency to feel disfavored or favored compared to their siblings, respectively. Given that parents employing conditional regard in one context might not necessarily do so in others, conditional regard is conceptualized in a domain‐specific way (Assor et al., 2004). In this study, we focused on parental conditional regard in the emotional and academic domains, which have been explored most often in previous research.

To account for the interdependence and complexity of sibling dyads, we employed the Actor‐Partner Interdependence Model (Cook & Kenny, 2005). This allowed us to investigate the agreement of siblings on parental conditional regard and the sibling relationship facets. We also considered the documented birth order effects on sibling comparison, differential parental affection, and conflict (Jensen et al., 2015; Jensen & McHale, 2017) and their potential effect on the relations investigated herein. Specifically, we investigated whether effects are distinguishable or indistinguishable for older and younger siblings, i.e., whether siblings are differentially affected by parental conditional regard. Furthermore, we examined the pattern of dyadic results (Kenny & Ledermann, 2010), including whether children's perceptions of their sibling relationships were influenced not only by their own experiences of parental conditional regard (actor effects) but also by their siblings' perception of it (partner effects).

We considered relevant background variables based on their established relevance in sibling research. Age and sibship size shape sibling roles and interaction patterns, influencing dimensions such as rivalry and warmth (Buist et al., 2013; McHale et al., 2012). Academic achievement serves as an indicator of child adjustment and has been linked to differential parental treatment and sibling comparisons (Jensen & McHale, 2015). Maternal education reflects broader socioeconomic resources and is associated with parenting behaviors that structure the family environment, including sibling dynamics (Weis et al., 2023). Finally, child gender was included as a fixed factor due to consistent evidence of gender patterns in sibling relationships (Updegraff et al., 2002).

## METHODS

### Participants and procedure

Participants were 201 sibling dyads. Data collection was part of a larger project funded by the Deutsche Forschungsgemeinschaft (DFG, German Research Foundation). Sample size was determined by a‐priori power analysis for the overall project. This study was not preregistered. We collected data from families with a target child aged 13–14 (or eighth grade), who had at least one sibling. We contacted families in Germany by letter via nine residents' registration offices and via schools to participate in an online survey. Families received 50 euros as monetary compensation if one parent, the child, and the age closest sibling participated in the study. Parents provided active informed consent; participation was voluntary. For the current study, we used all cohabiting sibling dyads. Families mostly had two (56.7%) or three (24.4%) children. We split children by age. The younger siblings' age ranged between 6 and 15 years (*M* = 12.21, *SD* = 1.74), and the older siblings' age ranged between 13 and 29 years (*M* = 15.29, *SD* = 2.31). Age difference between the participating siblings was on average 3.08 years (*SD* = 1.90). 51.7% of the younger siblings and 47.8% of the older siblings were female. Just over half of the dyads were same sex, with an almost even split between both girls (25.4%) and both boys (25.9%). Within the opposite sex dyads, slightly more dyads had an older brother (26.4%) than an older sister (22.4%).

### Measures

#### Parental conditional regard

Parental conditional regard was measured using a validated German adaptation (Otterpohl et al., 2017) of the Parental Conditional Regard Scale (Roth et al., 2009). Both siblings reported on their perception of conditional regard on a 7‐point Likert scale, ranging from 1 (not true at all) to 4 (neither nor) to 7 (exactly true).

Eight items were used for Parental Academic Conditional Positive Regard (PACPR). An example item is “When (or if) I get a very good grade at school, I feel that my mom loves me more”. For Parental Academic Conditional Negative Regard (PACNR), nine items were included such as “When (or if) I do not study hard—I feel that my mom appreciates me less”. Previous studies have demonstrated good validity and reliability for the scales (Otterpohl et al., 2017; Roth et al., 2009). Cronbach's alphas for this study were excellent for both PACPR (*α*_older_ = .91, *α*_younger_ = .90) and PACNR (*α*_older_ = .94, *α*_younger_ = .94) in older and younger siblings respectively.

Parental Emotional Conditional Positive Regard (PECPR) and Parental Emotional Conditional Negative Regard (PECNR) were each measured with respect to anger regulation. As the expression of anger is often least socially desired and for many parents difficult to handle, we focused on this emotion. Items for PECPR ask for parental reactions to a successful suppression of anger: “When I am angry at something or someone, but able to conceal it, I feel that my mother shows more interest in me than usual”. Items for PECNR ask for parental reactions when children express their anger: “When I am angry at something or someone and show it, I feel that my mother pays less attention to me than usual”. Otterpohl et al. (2017) demonstrated good validity, reliability and internal consistency for the emotional domain scales. Cronbach's alphas for the present study were excellent for both PECPR (*α*_older_ = .90, *α*_younger_ = .88) and PACNR (*α*_older_ = .92, *α*_younger_ = .91) in older and younger siblings respectively.

#### Sibling relationship

We used items based on the German version (Bojanowski et al., 2015) of the sibling relationship questionnaire (SRQ; Furman & Buhrmester, 1985). Due to the large project, only a selection of items was used, resulting in two to three items per scale. We used a 7‐point Likert scale, ranging from 1 (not true at all) to 4 (neither nor) to 7 (exactly true). Higher scores indicate higher levels of perceived competition, feeling (dis‐)favored, and conflict.

The two items “My brother/my sister and I compete with each other” and “My brother/my sister and I try to outdo each other in almost everything we do” measured sibling competition. Correlation between items was *r* = .80 for both older and younger siblings respectively.

The items “Usually my mother gives my brother/my sister more affection and attention than she gives me” and “Usually my mother prefers my brother/my sister over me” ask for children's perception or feeling of being disfavored by the mother. Parallel items ask for children's perception or feeling that they are favored by the mother. Correlation between items were satisfactory for feeling favored (*α*_older_ = .78, *α*_younger_ = .73) and feeling disfavored (*α*_older_ = .94, *α*_younger_ = .93).

Three items measured the extent of sibling antagonism and conflict (e.g., “My brother/my sister and I fight a lot.”). Cronbach's alphas were αs = .84 for older and younger siblings.

### Plan of analysis

We used the Actor‐Partner Interdependence Mediation Model (APIMeM) to test our hypotheses. Analyses were performed in Mplus 8 (Muthén & Muthén, 1998–2017). APIMeM extends simple mediation models through considering both siblings simultaneously within one model and modeling their interdependence. Specifically, the model includes actor or intrasibling effects, which refer to the effects of one sibling's variable on another variable of that same sibling, as well as partner or intersibling effects, which refer to the effect of one sibling's variable on a variable of the other sibling. The amount of missing data was minimal, ranging from 0% to 2.5% for the different variables. Little's Missing Completely at Random test (MCAR, Little, 1988) indicated that these data were missing completely at random (*χ* ^2^[38] = 24.45, *p* = .957). Therefore, we used the full information maximum likelihood procedure within MPlus.

We took a stepwise approach. In the first step, we created simpler and cleaner models using simple APIMeMs (see Figure S1). In the simple APIMeMs, we modeled the effect of parental conditional regard on sibling conflict, mediated by competition or feeling (dis‐)favored. For parental conditional regard, we alternated (1) between parental conditional positive and negative regard, and (2) between the academic and emotional domain. Additionally, we alternated (3) the mediators (competition, feeling favored and feeling disfavored), resulting in 2 × 2 × 3 simple APIMeMs (see Data S1 for the syntax). We followed the recommendations of Kenny and Ledermann (2010) in conducting the analyses. For each simple APIMeM we first calculated a saturated model, in which all paths were allowed. To examine indistinguishability of the effects between siblings we simultaneously fixed all actor and partner effects to be equal between the older and younger sibling (restricted model). We chose the restricted over the saturated model and therefore accepted indistinguishability if the *p*‐value of the chi‐square model difference test was > .20. This liberal alpha is used to avoid Type II error, ensuring that potential differences between older and younger siblings are not overlooked (Kenny & Ledermann, 2010). If the *p*‐value of the chi‐square test was below .20, we tested which paths needed to be unrestricted to receive a non‐significant difference to the saturated model, and treated those paths as distinguishable. This resulted in models with one degree of freedom less than the completely restricted model.

**FIGURE 1:**
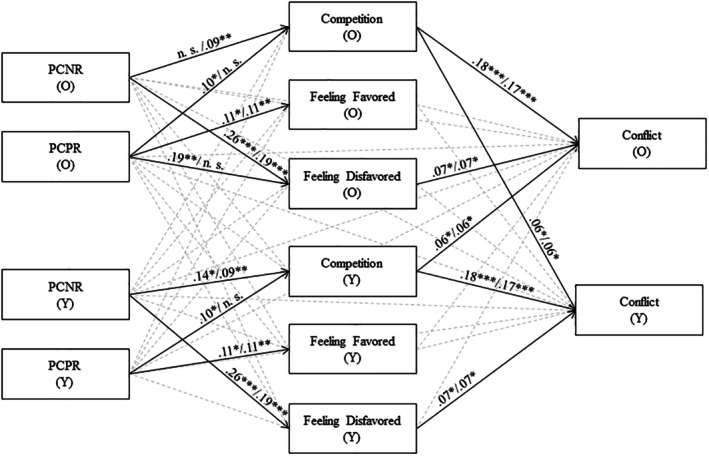
Overall Actor‐Partner Interdependence Mediation Model (APIMeM) for the effects of parental conditional regard on sibling conflict, considering competition and feeling favored/disfavored as mediators. Depicted are significant standardized regression coefficients for the model with parental conditional regard in the academic/emotional domain. Dashed, gray arrows indicate non‐significant paths. For parsimony, residuals and their correlations, as well as control variables are not displayed. PCNR, parental conditional negative regard; PCPR, parental conditional positive regard. O, older sibling; Y, younger sibling. ***p < .001. **p < .01. *p < .05.

In the second step, we combined all simple APIMeMs into two overall models for the academic and emotional domain, respectively, to allow for a more comprehensive interpretation. In parallel to the simple APIMeMs, we calculated a fully identified model and a model in which we restricted all actor and partner effects to be equal between older and younger siblings. If the restricted model differed significantly from the saturated model, we tested which paths needed to be unrestricted to receive a non‐significant difference to the saturated model and treated those paths as distinguishable in our final models (see Figure 1). Given that the path models of the APIMeMs included two parallel mediators, we used the Schoemann et al. (2017) Monte Carlo power analysis. Assuming a moderate effect size (.30 in a correlation metric), the power to detect a significant indirect effect in the current sample was 0.88. We present findings of the overall models in the manuscript. All findings and estimates of the simple APIMeMs (Tables S2–S4) and the overall model (Tables S5–S7) are presented in the Data S2. For transparency, we also present all simple APIMeMs and overall models without control variables in the Data S2 (see Tables S8–S11).

We tested indirect effects using the model constraint command in Mplus. The APIMeM allows estimating the indirect effects of actor effects only (actor–actor) as well as indirect effects involving partner effects (actor–partner, partner–actor, partner–partner). We examined indirect effects only when corresponding direct paths were significant.

In all analyses, we determined statistical significance of unstandardized effects (*b*) using 95% confidence intervals (CI, bootstrap method, *k* = 5000 samples) and *p*‐values. We have standardized the effects in the simple APIMeMs (see Table S3) and the final overall models (see Figure 1 and Table S5–S7) for better interpretation. We used the pooled *SD*s of older and younger siblings for the standardization of all effects that were equal for both siblings. We used younger or older siblings' *SD*s if effects differed for older and younger siblings.

To test for possible effects of background variables, we conducted MANCOVAs separately for older and younger siblings, accounting for data dependency. In each MANCOVA, the age of the child, academic achievement (mean grade from the last report), the number of siblings within the family, and maternal educational level were entered as covariates, and the gender of the child was entered as a fixed factor. Significant control variables were included in the APIMeMs.

## RESULTS

### Descriptive statistics and correlations

Means and standard deviations for the study variables separately for older and younger siblings, as well as mean‐level similarities (correlations) and differences (paired‐samples *t*‐tests) between siblings for the study variables are shown in Table 1. Similarities for PACNR, PECNR, and PECPR were low; siblings' reports were most similar for PACPR. With regard to sibling relationship variables, ratings were most similar for conflict, followed by competition. Agreement on feeling favored and disfavored was only small. Intrasibling correlations of feeling favored and disfavored showed medium high positive correlations, whereas siblings' agreement that the older sibling is the favored one, that is, intersibling correlations between the older sibling's report of being favored and the younger sibling's report of being disfavored, and vice versa, were low. Older siblings reported slightly higher PECPR and PECNR. Younger siblings were more likely to report receiving more affection from their mother than their sibling, whereas older siblings were more likely to report receiving less maternal affection than their younger counterpart.

**TABLE 1:** Descriptive statistics, sibling similarity (Pearson correlation) and differences for all study variables.

	Older siblings		Younger siblings		*r*	*d*	*t*_198‐200_	*p*(*t*)
	*M*	*SD*	*M*	*SD*				
PACNR academic	1.89	1.23	1.72	1.13	.18**	0.12	1.69	.093
PACPR academic	2.95	1.45	2.88	1.43	.34**	0.04	0.54	.589
PECNR emotional	2.82	1.69	2.25	1.49	.15*	0.27	3.87	.000
PECPR emotional	3.05	1.58	2.66	1.53	.16*	0.19	2.72	.007
Competition	3.28	1.83	3.23	1.86	.31**	0.02	0.25	.804
Feeling favored	2.02	1.36	2.38	1.61	.10	−0.17	−2.35	.020
Feeling disfavored	2.45	1.75	1.94	1.42	.14*	0.27	3.68	<.001
Conflict	3.67	1.68	3.74	1.74	.56**	−0.05	−0.67	.502

*Note*: *d* = standardized effect size (Cohen's *d*); *t* = paired‐samples *t*‐test.

Abbreviations: PACNR, parental academic conditional negative regard; PACPR, parental academic conditional positive regard; PECNR, parental emotional conditional negative regard; PECPR, parental emotional conditional positive regard.

* *p* < .05.

** *p* < .01.

Table 2 presents two types of correlations between study variables: intrasibling correlations, which represent the mean‐level correlations of older and younger siblings' reports separately, and intersibling correlations, which represent the mean‐level correlations between the older and younger siblings' reports. Intrasibling correlations between parenting and sibling variables were small to medium. Competition correlated with PCNR and with PCPR only in younger siblings. Feeling favored correlated more with PCPR, while feeling disfavored correlated more with PCNR. Conflict correlated with PACNR and PACPR, but not with PECPR, and with PECNR only in older siblings. Sibling relationship variables correlated positively, with two exceptions: the correlations of competition and feeling favored in older siblings, and feeling disfavored and conflict in younger siblings. Intersibling correlations showed that older siblings' perception of parental conditional regard is related to younger siblings' report of sibling relationship facets, with competition being associated with PECPR and PECNR, feeling favored being associated with PACPR, and conflict being associated with PACNR, PACPR, and PECNR. In other words, if the older sibling reported higher parental conditional regard than other older siblings, the younger sibling reported higher competition, feeling favored, and conflict than other younger siblings. In contrast, younger siblings' report of conditional regard showed little relationship to older siblings' perception of sibling relationship quality. Only older siblings' report of feeling favored was positively related to younger siblings' report of PACNR and PACPR. Further, one sibling's report on competition was related to the other sibling's report of conflict.

**TABLE 2:** Correlations (Pearson) between the study variables.

		(1)	(2)	(3)	(4)	(5)	(6)	(7)	(8)
Intrasibling correlations									
(1)	PACNR academic	–	.64**	.43**	.27**	.26**	.26**	.33**	.22**
(2)	PACPR academic	.64**	–	.38**	.41**	.25**	.24**	.22**	.18*
(3)	PECNR emotional	.52**	.36**	–	.34**	.21**	.16*	.24**	.10
(4)	PECPR emotional	.28**	.43**	.34**	–	.21**	.24**	.16*	.14
(5)	Competition	.16*	.13	.19**	.04	–	.24**	.22**	.33**
(6)	Feeling favored	.23**	.31**	.20**	.25**	.05	–	.48**	.19**
(7)	Feeling disfavored	.45**	.43**	.40**	.19**	.19**	.46**	–	.14
(8)	Conflict	.23**	.27**	.19**	.10	.44**	.15*	.22**	–
Intersibling correlations									
(1)	PACNR academic	.18**	.35**	.11	.23**	.02	.20**	.02	.11
(2)	PACPR academic	.22**	.34**	.11	.21**	.06	.15*	.08	.12
(3)	PECNR emotional	.10	.19**	.15*	.19**	−.02	.06	−.05	.04
(4)	PECPR emotional	.14	.20**	.15*	.16*	.07	.03	.03	.10
(5)	Competition	.14	.08	.18*	.18*	.31**	.10	.04	.18*
(6)	Feeling favored	.10	.15*	.06	.04	.16*	.10	.17*	.07
(7)	Feeling disfavored	.04	.11	.07	.11	.01	.27**	.14*	−.01
(8)	Conflict	.16*	.25**	.10	.19**	.24**	.08	.08	.56**

*Note*: Intrasibling correlations represent the correlations of all study variables reported by the older siblings below the diagonal and reported by the younger siblings above the diagonal. Intersibling correlations represent the correlations between all study variables reported by the older siblings (columns) and the younger siblings (rows), with the diagonal representing similarity in reports between siblings.

Abbreviations: PACNR, parental academic conditional negative regard; PACPR, parental academic conditional positive regard; PECNR, parental emotional conditional negative regard; PECPR, parental emotional conditional positive regard.

* *p* < .05.

** *p* < .01.

### Preliminary analyses

In the MANCOVA for older siblings, results revealed multivariate effects only for gender (Wilk's Lambda = .841, *F*[8, 156] = 3.696, *p* < .001) and maternal educational level (Wilk's Lambda = .858, *F*[8, 156] = 3.220, *p* = .002). Boys scored higher on competition (*d* = .41) and feeling favored (*d* = .33). Higher levels of maternal education were associated with lower levels of PECNR (*r* = .20, *p* < .01) and lower levels of conflict (*r* = −.17, *p* < .05) among older siblings. For younger siblings, results revealed multivariate effects for gender (Wilk's Lambda = .869, *F*[8, 158] = 2.980, *p* = .004), age (Wilk's Lambda = .893, *F*[8, 158] = 3.139, *p* = .003), grades (Wilk's Lambda = .901, *F*[8, 158] = 2.166, *p* = .033), and maternal educational level (Wilk's Lambda = .866, *F*[8, 158] = 3.068, *p* = .003). Younger brothers reported more PACPR (*d* = .37), PACNR (*d* = .27), PECPR (*d* = .28), competition (*d* = .35), and feeling favored (*d* = .47) compared with younger sisters. Younger siblings reported more conflict the younger they were in age (*r* = −.23, *p* < .01), the poorer their grades (*r* = .18, *p* < .05), and the lower their mothers' educational level (*r* = −.30, *p* < .01). Based on the MANCOVA findings, these background variables were included as covariates in the following APIMeMs. Partial correlations that control for these background variables are presented in the Data S2 (Table S1). Differences between correlations with and without control variables were non‐significant, ranged between −.08 < *r*_**Δ**_ < .09, and were smaller than *r*_**Δ**_ < .05 in 87.5%. Changes in intrasibling correlations were largest for older siblings' PACPR and competition (*r*_**Δ**_ = .09), as well as correlations of younger siblings PECNR with feeling disfavored (*r*_**Δ**_ = −.06) and conflict (*r*_**Δ**_ = −.07). Changes in intersibling correlations were largest for correlations of younger siblings' PECNR with older siblings PECNR (*r*_**Δ**_ = −.08) and PECPR (*r*_**Δ**_ = −.07), as well as younger siblings' PACPR with older siblings feeling favored and disfavored, younger siblings' feeling favored and older siblings' competition, and conflict between the two siblings (*r*_**Δ**_ = −.06).

### Simple actor‐partner interdependence mediation model

The results of the simple Actor‐Partner Interdependence Mediation Model (APIMeM) revealed that, in most cases, the restricted models did not significantly differ from the saturated model (see Table S2), indicating that the effects of parental conditional regard on sibling relationships were indistinguishable between siblings.

The path coefficients of the simple APIMeMs are presented in the Data S2 (Table S3). Significant actor effects were found for all types of conditional regard on the three mediators (competition, feeling favored, and feeling disfavored). Effects were slightly higher for conditional regard in the academic domain compared to the emotional domain. Notably, PACPR had a higher actor effect on feeling disfavored for older than for younger siblings, resulting in the only distinguishable paths in all simple APIMeMs. Furthermore, partner effects were found from PACNR on feeling favored and from PECPR on competition, indicating that one sibling's perception of parenting predicted the other sibling's report of sibling relationship. Indirect effects revealed that competition and feeling disfavored, but not feeling favored, mediated the effects of parental conditional regard on conflict (see Table S4). Additionally, PACNR and PACPR showed an indirect actor‐partner effect on conflict through competition, meaning that PACNR and PACPR had an actor effect on competition, which in turn had a partner effect on conflict. PACNR was still directly related to conflict in all models. For PACPR and PECNR, only the direct effects in the models with feeling favored as a mediator were significant, suggesting that feeling favored is not a mediator in these models. PECPR did not have a direct association with conflict in any of the models and no indirect association through competition, which is consistent with the non‐significant correlations.

### Overall actor‐partner interdependence mediation model

The academic model, wherein all actor and partner effects were constrained to be equal, fitted significantly worse than the unrestricted model, *χ* ^2^(22) = 29.261, *p* = .138; *CFI* = .984, *RMSEA* = .041, *SRMR* = .028. Unrestricting two paths resulted in a model with no significant difference from the saturated model, *χ* ^2^(20) = 15.264, *p* = .761; *CFI* = 1.000, *RMSEA* = .000, *SRMR* = .020. Specifically, the actor effect of PACPR on feeling disfavored and the actor effect of PACNR on competition were unrestricted. In the emotional domain, the model where all actor and partner effects were set equal did not differ significantly from the saturated model, *χ* ^2^(22) = 24.946, *p* = .300; *CFI* = 0.993, *RMSEA* = .026, *SRMR* = .027. Final models are depicted in Figure 1; unstandardized effects, standardized effects, standard error, and confidence intervals of all estimates are presented in the Data S2 (Tables S4 and S5); estimates for all control variables are presented in the Data S2 (Tables S6 and S7).

The overall models showed that PACPR and PECNR are related to competition, whereas PACNR only related to competition in younger siblings. PACPR and PECPR were related to feeling favored, while PACNR and PECNR were related to feeling disfavored. The effect of PECNR was slightly smaller in magnitude than the effect of PACNR. Additionally, PACPR related to feeling disfavored in older siblings. All effects of conditional regard were actor effects. Competition and feeling disfavored showed significant actor effects on siblings conflict. In addition, the partner effect of competition on conflict was significant. Direct effects of parental conditional regard on conflict were not significant in the overall models.

The indirect actor‐actor effect of PACPR on conflict via competition was significant (*b* = .05; 95% CI: 0.01–0.11, SE = .03, *p* = .030). Also, PECNR showed an indirect actor‐actor effect on conflict via competition (*b* = .05; 95% CI: 0.01–0.09, SE = .02, *p* = .017). The indirect actor‐actor effects of PACNR (*b* = .05; 95% CI: 0.00–0.11, SE = .03, *p* = .017) and PECNR (*b* = .04; 95% CI: 0.00–0.08, SE = .02, *p* = .070) via feeling disfavored were only marginally significant. The indirect actor‐actor effect of younger siblings' PACNR via competition (*b* = .08; 95% CI: −0.00–0.17, SE = .04, *p* = .057) and the actor‐partner effect of PACPR (*b* = .02; 95% CI: 0.00–0.04, SE = .01, *p* = .086) was also marginally significant.

Background variables showed significant relations with study variables (Table S3). Maternal educational level, younger siblings' age, and grade showed (if any) only small significant associations ranging from .07 < *β* < .16. However, gender showed rather high actor effects on competition (*β*_O_ = −.42, *p*_O_ = .002; *β*_Y_ = −.29, *p*_Y_ = .046) and feeling favored (*β*_O_ = −.28, *p*_O_ = .040; *β*_Y_ = −.36, *p*_Y_ = .012). Further, younger siblings' gender had a significant partner effect on older siblings competition (*β* = −.35, *p* = .014). The direction of all these effects was that male gender was associated with higher competition and feeling favored.

## DISCUSSION

The impact of parental conditional regard on children's individual motivational‐emotional development and their ability to form close relationships is well documented (Assor et al., 2004; Kanat‐Maymon et al., 2023; Roth et al., 2009). The purpose of our study was to investigate how parental conditional regard relates to various facets of the sibling relationship, a significant familial bond beyond parent–child interactions. In doing so, we addressed the different perceptions of siblings on parenting and whether effects are generalizable or distinguishable for older and younger siblings.

We add to the literature by providing initial evidence that parental conditional regard comes with detrimental effects for the sibling relationship. Specifically, if siblings perceived parental conditional regard, they also reported higher competition, favoritism, and conflict in their sibling relationship. These findings align with various findings showing detrimental effects for the individual child and support the assumptions of self‐determination theory that parental conditional regard represents a parenting style that undermines children's capacity to build high‐quality social relationships (Kanat‐Maymon et al., 2023). Beyond emotional capacities (Roth & Assor, 2012), our results suggest that sibling competition and perceptions of being disfavored play significant roles as potential mechanisms through which parental conditional regard affects children's relationship quality. Effects were generalizable across parental conditional regard in the academic and emotional domain, although effects were a bit more pronounced in the academic domain. This slight disparity may be attributed to the fact that grades are typically more salient and easier to compare between siblings in contrast to success in emotion control. As a result, the impact of conditional regard for academic performance may be more impactful for sibling dynamics.

Our findings also revealed only low to moderate agreement in siblings' perceptions of parental conditional regard. This suggests that parental conditional regard more accurately reflects a nonshared environment among siblings. These findings align with other literature demonstrating rather low to moderate levels of agreement with regard to parenting (van der Kaap‐Deeder et al., 2015), fairness of differential treatment (Kowal et al., 2006) or sibling relationships in general (Lecce et al., 2009), although other literature suggests higher agreement on general measures of sibling relationship quality (*r* = .71; Barberis et al., 2022). Yet, our findings align with the broader debate on the relative influence of genetics versus environment, providing substantial evidence that most environmental influence, for example, with regard to personality, is nonshared (Plomin & Daniels, 2011). Whether the divergent views of siblings arise from actual differential parenting practices or differences in the perception of the same behaviors warrants further investigation.

Interestingly, siblings' perceptions of parental conditional regard predominantly related to their own perception of sibling relationship quality rather than their siblings' views, and effects were generally equal for older and younger siblings. This aligns with research emphasizing that it is a child's perception of controlling parenting that predicts their adjustment (Mabbe et al., 2016). Notably, while sibling conflict (e.g., quarrels) showed rather high similarity between siblings, other facets such as competition and feelings of favoritism showed lower agreement, implying they may reflect internal representations of the sibling relationship. Parental conditional regard may shape children's perceptions in ways that negatively impact their perception of the sibling relationship. This interpretation resonates with research indicating that individuals who perceive conditional regard from parents tend to project such perceptions onto others (Moller et al., 2019). Ultimately, it underlines the importance of using children's perceptions of conditional regard and methods that acknowledge these different perspectives, such as APIMeM (Cook & Kenny, 2005).

### Parental conditional regard and sibling competition

Our results support our assumption that perceived parental conditional regard fosters children's perceptions of being in competition with their sibling. Within evolutionary psychology, evidence suggests that siblings can be both rivals or resources, depending on various factors, among others, gender or age spacing (Pollet & Hoben, 2012). Our results suggest that providing a need‐frustrating setting might be one important contextual condition fostering rivalry and competition for limited resources. Providing support for children's basic psychological needs is crucial to create a more resourceful relation between siblings (van der Kaap‐Deeder et al., 2015).

Particularly PACPR relates to competition in both siblings. This finding is consistent with previous research suggesting that providing a surplus of affection and regard, that is, providing PACPR, can lead to competitive mindsets in children (Roth et al., 2009; Steffgen et al., 2022). Our results add to this literature by showing that this effect is not limited to children's personal adjustment and motivation, but also manifests within the sibling relationship as competition. Furthermore, the withholding of affection after school failures, that is, PACNR, is related to higher competition. This effect was observed consistently for both siblings in the simple APIMeMs, but only in younger siblings in the overall model. Younger siblings have a higher orientation toward social comparisons (Jensen et al., 2015). In conditionally regarding settings, this comparison orientation may particularly lead younger siblings to develop more competition as they try to outdo their sibling to gain parental regard. Yet, considering the differing results between simple APIMeMs and the overall model, further research is needed to establish robust conclusions on whether PCNR promotes sibling competition differentially for older and younger siblings. Also, given the high correlation between PCNR and PCPR, particularly in the academic domain, including both in the model leads to competition for shared variance. As a result, the unique variances may not be as impactful.

In the emotional domain, PECNR was related to competition, whereas PECPR was not. This distinction may be attributed to the fact that PECNR promotes dysregulation, whereas PECPR promotes emotional suppression (Roth et al., 2009). While dysregulation might exacerbate competition and conflict, emotional suppression may actually lead siblings to avoid these behaviors. However, as suppression reduced the ability to disclose negative feelings in romantic couples (Roth et al., 2009), PECPR potentially affects other facets of sibling relationships such as closeness and intimacy, which should be addressed in future studies. An alternative explanation comes from Prospect Theory (Kahneman & Tversky, 1979), which suggests that individuals tend to weigh potential losses more heavily than potential gains. This may explain why PCNR compared to PCPR in general has more negative effects (Otterpohl et al., 2017, 2020). Specifically, losses resulting from PECNR, particularly in terms of basic psychological need satisfaction, may motivate siblings more strongly to regain need satisfaction through competing.

Beside parental conditional regard, gender was a strong predictor of competition, which is consistent with existing literature on gender differences (Jensen et al., 2015). This may be due to the broad measure of competition employed in our study. We assume that parental conditional regard may be stronger related to competition when measured domain‐specifically, as research suggests that effects are typically stronger for domain‐specific outcomes (Roth et al., 2009). Future research should investigate whether parental conditional regard particularly promotes sibling competition in domains where siblings perceive parental conditional regard. Additionally, considering the role of social reference norm may also strengthen the predictive power of conditional regard. Currently, conditional regard is measured as an individual norm. However, in the sibling context, a measurement with a social norm, such as “When I perform better than my sibling…”, may provide additional insights. This type of conditional regard may be more strongly associated with competition.

### Parental conditional regard and feelings of being (dis‐)favored

Consistent with our hypothesis, PCNR was primarily associated with feelings of being disfavored, while PCPR was associated with feelings of being favored. These findings support our hypothesis that the frustrated or diluted basic psychological needs created through PCNR lead to a sense of devaluation and neediness in children (Assor & Tal, 2012), resulting in both siblings feeling short‐changed and disfavored. In contrast, the self‐aggrandizing effect of PCPR (Assor & Tal, 2012) manifests as feelings of being favored within the sibling relationship.

Interestingly, our findings suggest that perceived (dis‐)favoritism among siblings not necessarily reflects an objective state, where parents provide more or less appreciation to one sibling, but rather a subjective experience. This interpretation is supported by the finding that siblings' reports of feeling disfavored and favored were more highly correlated within siblings (intrasibling) than that siblings agreed on which sibling is the favored or disfavored one (intersibling). Meta‐analyses reveal that traditionally, the measurement of favoritism relies on a single report from one sibling or parent, or on difference scores between siblings (Jensen, Jorgensen‐Wells, et al., 2023; Jensen & Jorgensen‐Wells, 2025) implicitly assuming that there is an underlying “true” favoritism within families. Our measurement approach suggests an additional nuanced understanding of perceived favoritism as involving a subjective experience, potentially shaped by frustrated and diluted basic psychological needs through parental conditional regard. Examination of each sibling's individual perception and identifying potential antecedents of these perceptions, such as parental conditional regard, can add a new facet to the current field of research on perceived favoritism.

The effects of feeling favored are more ambivalent. On the one hand, favoritism in general was found to impair the well‐being of the individual sibling and the sibling relationship; on the other hand, being slightly favored can be beneficial under certain circumstances and be perceived as fair (Gozu & Newman, 2020; McHale et al., 1995; Singer & Weinstein, 2000). Also, research in adult siblings suggests non‐linear relations, where feeling slightly favored can be beneficial, but excessive favoritism impairs sibling relationship quality, even for the favored child (Boll et al., 2003). These findings fit with the description of PCPR as a double‐edged sword, where the provision of appreciation for meeting external expectations may satisfy basic needs in the short term, leading to compliance, but ultimately results in diluted need satisfaction, performance flaws, and emotional costs (Kanat‐Maymon et al., 2023). In sum, while it might be beneficial to feel favored through PCPR to some extent, persistent or excessive perceived favoritism through PCPR potentially harms the sibling relationship.

In addition, PACPR was also associated with feeling disfavored among older siblings in the overall model. In the simple APIMeMs, this relationship existed for both siblings but was more pronounced among older siblings. Existing research indicates that younger siblings tend to receive more affection from their parents in general (Jensen & McHale, 2017). Accordingly, older siblings in our study reported feeling less favored but more disfavored in comparison to their younger siblings. The perception of a potentially objective discrepancy could be particularly pronounced and perceived as unfair if older siblings view parental love as uncertain, contributing to their feelings of being overlooked. This finding challenges the assumption that an oversupply of affection would naturally lead to feeling favored.

### Sibling conflicts

Aligning with our expectations, competition and feeling disfavored mediated the relationship between parental conditional regard and sibling conflicts. Our findings contribute to the understanding that need‐frustrating parenting not only impacts conflict resolution in sibling relationships (van der Kaap‐Deeder, 2021) but also serves as a catalyst for the emergence of quarrels in the first place. The indirect effects were significant in the simple APIMeMs for PACNR, PACPR, and PECNR, but only partly significant or marginally significant in the overall model. Further, while simple APIMeMs suggested only partial mediation from PCNR to conflict, the overall model indicated full mediation. In contrast, only PCPR in the academic domain related to conflict, but only in the simple APIMeM.

Quarrels between siblings may ultimately result from thwarted needs, as present when children feel disfavored or in competition with each other. It is argued that when competition stems from external pressures, it becomes need‐frustrating (Ryan & Reeve, 2021). Parental conditional regard likely imposes this external pressure, compelling siblings to compete for limited resources. These findings align with prior research indicating relations between basic need frustration and aggressive behavior (Campione‐Barr et al., 2014; Kuzucu & Şimşek, 2013). In addition, the analyses revealed a partner effect where one sibling's perception of competition was linked to the other sibling's perception of conflict. These intersibling relations suggest that both siblings' views on competition contribute to their individual perceptions of conflict, highlighting the interconnectedness of sibling dynamics. By intensifying competition in both siblings, parental conditional regard might have an amplified, potentially additive impact on sibling conflicts. Future studies should replicate the robustness of this effect and verify the potential additive impact of parental conditional regard.

### Limitations and future directions

We examined various facets of the sibling relationship to gain initial insights into potential negative effects of parental conditional regard. However, given the cross‐sectional nature of our data, caution is needed when drawing causal conclusions. For instance, reciprocal effects of parental conditional regard were found between parents and their adolescent children's contingent self‐esteem (Otterpohl et al., 2021). Sibling conflicts might lead parents to intervene with stricter rules, possibly leading to a more controlling parenting style in an effort to maintain order within the family. Also, sibling competition and favoritism are likely reciprocally interconnected. When siblings engage in comparisons and competition with each other, siblings may pay more attention to what the sibling and themselves receive; thus, favoritism may become particularly noticeable (Jensen et al., 2015). Further, Midgley et al. (2022) showed that siblings experience lower self‐evaluations when they are preoccupied with parental regard and recall instances where their sibling outperformed them. The consistently outperforming sibling may receive more affection, leading to feelings of being favored, while the outperformed sibling may feel particularly disfavored. But also, the perception of (dis‐)favoritism likely promotes competition, as the favored sibling may feel motivated to maintain this status of being preferred and the disfavored sibling may feel motivated to improve his status. Further longitudinal and experimental research is necessary to establish causality conclusively.

In APIMeMs, categorizing participants into two groups is essential. In sibling research, a common approach is to categorize siblings based on age (e.g. Barberis et al., 2022; van der Kaap‐Deeder et al., 2015). In our study, this led to a wide age range within both older and younger sibling groups, resulting in significant age‐related heterogeneity. While most effects could be generalized to older and younger siblings, some paths differed between the two groups. Utilizing a more homogeneous sample (e.g., ensuring all older siblings are the same age) could reveal more meaningful and age‐specific differences between siblings.

A third limitation concerns the measurement of sibling‐related variables, which resulted from the nature of the data as part of a larger research project. Consequently, the constructs of interest were assessed with a limited number of items, covering relatively narrow constructs. Future studies should utilize broader scales to capture these constructs more comprehensively, potentially leading to a more nuanced understanding of the effects under investigation. For instance, exploring domain‐specific outcomes, such as competition within each domain (Roth et al., 2009), could be valuable.

## CONCLUSION

Our study provides initial insights into the detrimental consequences of parental conditional positive and negative regard on various aspects of the sibling relationship. More specifically, our results indicate the important role of PCPR and PCNR for sibling competition, the upward and downward differentiation between PCPR and PCNR when considering feelings of being favored and disfavored, respectively, and the mediating role of competition and feelings of being disfavored for the effect of parental conditional regard on conflict. Using the Actor‐Partner Interdependence Mediation Model revealed overall low similarities in siblings' perceptions of parenting and the sibling relationship, the importance of actor effects over partner effects, as siblings' perceptions of conditional regard predominantly influence their own rather than their siblings' perceptions of sibling relationship quality, and the potential of cumulative effects through intersibling (partner) effects of competition on conflict.

The present study is relevant for practitioners aiming to improve sibling and parent–child relationships. It is crucial to educate parents about the potentially detrimental effects of conditional regard. Awareness of these consequences can lead to more informed parenting practices. This is particularly important for the seemingly benign form of conditional positive regard, as it may lead to more subtle negative effects. Furthermore, understanding sibling relationship quality through a constructivist lens rather than as solely objective is essential, especially concerning perceptions of parental conditional regard and favoritism. Instead of attempting to treat both siblings identically, it seems crucial to provide appropriate support for the basic psychological needs of each child. Overall, by reducing parental conditional regard and emphasizing need support and unconditional positive regard, parents can foster healthier sibling relationships, reduce conflict, and mitigate the adverse impacts of differential treatment.

## AUTHOR CONTRIBUTIONS

**Sarah Teresa Steffgen**: Conceptualization, methodology, formal analysis, data curation, writing—original draft preparation. **Nantje Otterpohl**: Conceptualization, funding acquisition, project administration, writing—reviewing and editing. **Avi Assor**: Conceptualization, funding acquisition, project administration. **Yaniv Kanat‐Maymon**: Conceptualization, funding acquisition, formal analysis. **Bat El Gueta**: Conceptualization. **Malte Schwinger**: Conceptualization, funding acquisition, project administration, supervision, writing—reviewing and editing. All authors commented on previous versions of the manuscript. All authors have read and approved the final manuscript.

## FUNDING INFORMATION

The study was supported by a grant from the Deutsche Forschungsgemeinschaft (DFG, German Research Foundation)—396850149.

## CONFLICT OF INTEREST STATEMENT

The authors have no conflicts of interest to declare relevant to the content of this article.

## ETHICS STATEMENT

The study was approved by the local ethics committee of the Department of Psychology, Justus‐Liebig University (Ethics approval number: 2016–0028).

## INFORMED CONSENT

Informed consent was obtained from all the participants of the study.

## Supporting information


Data S1:

Data S2:

## Data Availability

The data that support the findings of this study are available from the corresponding author upon reasonable request.
