# Supplementary material for: “They always disfavor me!”: Parental conditional regard undermines teenage sibling relationships through raising competition and perceived disfavoritism
Source: J Res Adolesc. 2025 Sep 8;35(3):e70071. doi: 10.1111/jora.70071 (PMC12416125; doi:10.1111/jora.70071)
Supplement: Supplementary file 1 — Data S1: [file JORA-35-0-s001.docx]

**Supplementary Materials**

**Syntax for the simple APIMeM**

VARIABLE:

missing = all(999);

names =

S1PACPR S1PACNR S1PECPR S1PECNR !older sibling parental conditional regard

S1COMP S1FAV S1DISFAV !older sibling competition, feeling favored, feeling disfavored

S1CONF !older sibling conflict

S2PACPR S2PACNR S2PECPR S2PECNR !younger sibling parental conditional regard

S2COMP S2FAV S2DISFAV !younger sibling competition, feeling favored, feeling disfavored

S2CONF !younger sibling conflict

;

usevar = S1PACNR S2PACNR ! vary between conditional positive and negative regard ! vary between conditional regard in the academic and emotional domain

S1COMP S2COMP !Vary between competition, feeling favored, feeling disfavored

S1CONF S2CONF;

ANALYSIS:

estimator = ml;

bootstrap = 5000;

MODEL:

!a-paths

S1COMP on S1PACNR (aA); !a-path actor effect

S2COMP on S2PACNR (aA);

S1COMP on S2PACNR (aP); !a-path partner effect

S2COMP on S1PACNR (aP);

!b-paths

S1CONF on S1COMP (bA); !b-path actor effect

S2CONF on S2COMP (bA);

S2CONF on S1COMP (bP); !b-path partner effect

S1CONF on S2COMP (bP);

!c-paths

S1CONF on S1PACNR (cdashA); !c-path partner effect

S2CONF on S2PACNR (cdashA);

S2CONF on S1PACNR (cdashP); !c-path partner effect

S1CONF on S2PACNR (cdashP);

!Correlating response and predictor variables to model inter-personal dyadic dependence

S1PACNR WITH S2PACNR;

S1COMP WITH S2COMP;

S1CONF WITH S2CONF;

OUTPUT: cinterval(bootstrap);
