# Supplementary material for: “They always disfavor me!”: Parental conditional regard undermines teenage sibling relationships through raising competition and perceived disfavoritism
Source: J Res Adolesc. 2025 Sep 8;35(3):e70071. doi: 10.1111/jora.70071 (PMC12416125; doi:10.1111/jora.70071)
Supplement: Supplementary file 2 — Data S2: [file JORA-35-0-s002.docx]

**Supplementary Materials**

Table S1. Partial correlations (Pearson) between the study variables.

|  | | (1) | (2) | (3) | (4) | (5) | (6) | (7) | (8) |
| --- | --- | --- | --- | --- | --- | --- | --- | --- | --- |
| **Intrasibling correlations** | |  |  |  |  |  |  |  |  |
| (1) | PACNR academic | – | .63*** | .37*** | .34*** | .22** | .31*** | .34*** | .15 |
| (2) | PACPR academic | .65*** | – | .37*** | .43*** | .24** | .25** | .23** | .14 |
| (3) | PECNR emotional | .56*** | .41*** | – | .38*** | .19** | .13 | .18* | .03 |
| (4) | PECPR emotional | .29*** | .45*** | .33*** | – | .17* | .20* | .19* | .15 |
| (5) | Competition | .17* | .22** | .20* | .07 | – | .24** | .26** | .38*** |
| (6) | Feeling favored | .19* | .30*** | .20* | .27*** | .08 | – | .55*** | .15 |
| (7) | Feeling disfavored | .46*** | .44*** | .44*** | .20* | .22** | .46*** | – | .17* |
| (8) | Conflict | .22** | .24** | .23** | .08 | .43*** | .16* | .26** | – |
| **Intersibling correlations** | |  |  |  |  |  |  |  |  |
| (1) | PACNR academic | .16* | .34*** | .08 | .19* | .02 | .18* | .01 | .08 |
| (2) | PACPR academic | .22** | .35*** | .11 | .16* | .09 | .21** | .14 | .13 |
| (3) | PECNR emotional | .07 | .17* | .07 | .12 | -.02 | .02 | -.09 | .00 |
| (4) | PECPR emotional | .14 | .25** | .19* | .19* | .02 | .06 | .03 | .12 |
| (5) | Competition | .14 | .13 | .22** | .22** | .27** | .10 | .04 | .17* |
| (6) | Feeling favored | .10 | .17* | .11 | .05 | .08 | .13 | .17* | .02 |
| (7) | Feeling disfavored | .02 | .13 | .04 | .12 | .02 | .31*** | .14 | -.02 |
| (8) | Conflict | .14 | .21** | .13 | .18* | .28*** | .09 | .09 | .50*** |

*Note.* PACPR = parental academic conditional positive regard; PACNR = parental academic conditional negative regard; PECPR = parental emotional conditional positive regard; PECNR = parental emotional conditional negative regard.

*** p < .001; ** p < .01. * p < .05.

Correlations are controlled for older and younger siblings’ gender, maternal educational level, younger siblings’ age and grades.

Intrasibling correlations represent the correlations of all study variables reported by the older siblings below the diagonal and reported by the younger siblings above the diagonal. Intersibling correlations represent the correlations between all study variables reported by the older siblings (colums) and the younger siblings (rows), with the diagonal representing similarity in reports between siblings.

Table S2. Fit indices of restricted simple APIMeM predicting sibling conflict by parental conditional regard, mediated by competition and feeling favored/disfavored.

|  | χ² | *df* | *p* | *CFI* | *RMSEA* | *SRMR* |
| --- | --- | --- | --- | --- | --- | --- |
| **PACNR** |  |  |  |  |  |  |
| Competition | 7.052 | 6 | .316 | .995 | .030 | .019 |
| Feeling favored | 2.542 | 6 | .864 | 1.000 | .000 | .012 |
| Feeling disfavored | 5.306 | 6 | .505 | 1.000 | .000 | .021 |
| **PACPR** |  |  |  |  |  |  |
| Competition | 6.648 | 6 | .355 | .997 | .023 | .019 |
| Feeling favored | 4.512 | 6 | .608 | 1.000 | .000 | .016 |
| Feeling disfavored | 13.497 | 6 | .036 | .958 | .079 | .032 |
| Feeling disfavored, specific | 4.347 | 5 | .501 | 1.000 | .000 | .016 |
| **PECPR** |  |  |  |  |  |  |
| Competition | 8.552 | 6 | .200 | .988 | .046 | .022 |
| Feeling favored | 2.624 | 6 | .854 | 1.000 | .000 | .011 |
| Feeling disfavored | 3.551 | 6 | .737 | 1.000 | .000 | .013 |
| **PECNR** |  |  |  |  |  |  |
| Competition | 8.395 | 6 | .211 | .989 | .045 | .023 |
| Feeling favored | 2.954 | 6 | .815 | 1.000 | .000 | .014 |
| Feeling disfavored | 8.468 | 6 | .206 | .986 | .045 | .027 |

*Note.* Restricted models are those, in which all actor and partner effects are set equal for older and younger siblings (df = 6). If *p* < .20 for the completely restricted models (bold values), we freed specific paths, to find the most restricted model, which is still not distinct from the fully identified model (df = 5).

PACPR = parental academic conditional positive regard; PACNR = parental academic conditional negative regard; PECPR = parental emotional conditional positive regard; PECNR = parental emotional conditional negative regard.

Analyses are controlled for the gender of both siblings, maternal educational level and the age and grades of the younger sibling.

Table S3. Simple APIMeMs predicting sibling conflict by parental conditional regard, mediated by competition and feeling favored/disfavored.

| **a-paths**  **Parenting 🡪 Mediator** | ***b*_O/Y_** | **95%-CI_O/Y_** | ***SE*_O/Y_** | ***p*_O/Y_** | **β_O/Y_** |
| --- | --- | --- | --- | --- | --- |
| **PACNR actor effects** |  |  |  |  |  |
| Competition | **0.27** | 0.13, 0.42 | 0.07 | .000 | .15 |
| Feeling favored | **0.25** | 0.12, 0.39 | 0.07 | .000 | .16 |
| Feeling disfavored | **0.55** | 0.39, 0.70 | 0.08 | .000 | .35 |
| **PACNR partner effects** |  |  |  |  |  |
| Competition | 0.03 | -0.13, 0.19 | 0.08 | .753 | .01 |
| Feeling favored | **0.14** | 0.01, 0.28 | 0.07 | .037 | .10 |
| Feeling disfavored | -0.05 | -0.16, 0.07 | 0.06 | .412 | -.03 |
| **PACPR actor effects** |  |  |  |  |  |
| Competition | **0.25** | 0.14, 0.39 | 0.05 | .000 | .13 |
| Feeling favored | **0.23** | 0.13, 0.34 | 0.05 | .000 | .16 |
| Feeling disfavored | **0.55/**  **0.23** | 0.36, 0.71/  0.09, 0.38 | 0.09/  0.07 | .000/  .002 | .31/  .16 |
| **PACPR partner effects** |  |  |  |  |  |
| Competition | -0.00 | -0.12, 0.16 | 0.06 | .965 | .00 |
| Feeling favored | 0.08 | -0.03, 0.18 | 0.05 | .145 | .05 |
| Feeling disfavored | -0.01 | -0.12, 0.12 | 0.06 | .902 | -.01 |
| **PECNR actor effects** |  |  |  |  |  |
| Competition | **0.20** | 0.10, 0.33 | 0.06 | .000 | .11 |
| Feeling favored | **0.15** | 0.07, 0.24 | 0.05 | .001 | .10 |
| Feeling disfavored | **0.33** | 0.22, 0.45 | 0.06 | .000 | .21 |
| **PECNR partner effects** |  |  |  |  |  |
| Competition | 0.05 | -0.05, 0.19 | 0.06 | .385 | .03 |
| Feeling favored | 0.05 | -0.04, 0.15 | 0.05 | .281 | .03 |
| Feeling disfavored | 0.03 | -0.11, 0.05 | 0.04 | .499 | .02 |
| **PECPR actor effects** |  |  |  |  |  |
| Competition | 0.10 | -0.02, 0.22 | 0.06 | .096 | .05 |
| Feeling favored | **0.21** | 0.12, 0.30 | 0.05 | .000 | .14 |
| Feeling disfavored | **0.16** | 0.06, 0.26 | 0.05 | .002 | .10 |
| **PECPR partner effects** |  |  |  |  |  |
| Competition | **0.12** | 0.01, 0.23 | 0.06 | .043 | .06 |
| Feeling favored | -0.01 | -0.10, 0.09 | 0.05 | .924 | .00 |
| Feeling disfavored | 0.07 | -0.03, 0.17 | 0.05 | .198 | .04 |
| **b-paths**  **Mediator 🡪 Conflict** |  |  |  |  |  |
| **PACNR actor effects** |  |  |  |  |  |
| Competition | **0.32** | 0.23, 0.40 | 0.04 | .000 | .19 |
| Feeling favored | 0.10 | -0.01, 0.21 | 0.06 | .076 | .06 |
| Feeling disfavored | **0.17** | 0.07, 0.28 | 0.05 | .001 | .10 |
| **PACNR partner effects** |  |  |  |  |  |
| Competition | **0.10** | 0.02, 0.17 | 0.04 | .011 | .06 |
| Feeling favored | 0.03 | -0.08, 0.14 | 0.06 | .574 | .02 |
| Feeling disfavored | -0.02 | -0.13, 0.09 | 0.06 | .713 | -.01 |
| **PACPR actor effects** |  |  |  |  |  |
| Competition | **0.32** | 0.24, 0.43 | 0.04 | .000 | .19 |
| Feeling favored | 0.11 | -0.01, 0.21 | 0.06 | .058 | .06 |
| Feeling disfavored | **0.18** | 0.08, 0.28 | 0.05 | .000 | .11 |
| **PACPR partner effects** |  |  |  |  |  |
| Competition | **0.10** | -0.02, 0.20 | 0.04 | .014 | .06 |
| Feeling favored | 0.03 | -0.09, 0.14 | 0.06 | .590 | .02 |
| Feeling disfavored | -0.02 | -0.12, 0.09 | 0.05 | .721 | -.01 |
| **PECNR actor effects** |  |  |  |  |  |
| Competition | **0.32** | 0.23, 0.41 | 0.04 | .000 | .19 |
| Feeling favored | **0.12** | 0.01, 0.22 | 0.05 | .033 | .07 |
| Feeling disfavored | **0.19** | 0.08, 0.29 | 0.05 | .000 | .11 |
| **PECNR partner effects** |  |  |  |  |  |
| Competition | **0.10** | 0.02, 0.18 | 0.04 | .016 | .06 |
| Feeling favored | 0.05 | -0.06, 0.15 | 0.05 | .390 | .03 |
| Feeling disfavored | -0.01 | -0.11, 0.09 | 0.05 | .831 | -.01 |
| **PECPR actor effects** |  |  |  |  |  |
| Competition | **0.33** | 0.24, 0.41 | 0.04 | .000 | .19 |
| Feeling favored | **0.13** | 0.02, 0.23 | 0.05 | .018 | .07 |
| Feeling disfavored | **0.20** | 0.10, 0.29 | 0.05 | .000 | .12 |
| **PECPR partner effects** |  |  |  |  |  |
| Competition | **0.10** | 0.02, 0.17 | 0.04 | .014 | .06 |
| Feeling favored | 0.04 | -0.07, 0.15 | 0.06 | .497 | .02 |
| Feeling disfavored | -0.00 | -0.10, 0.09 | 0.05 | .942 | .00 |
| **c-paths**  **Parenting 🡪 Conflict** |  |  |  |  |  |
| **PACNR actor effects** |  |  |  |  |  |
| Competition | **0.15** | 0.03, 0.27 | 0.06 | .018 | .09 |
| Feeling favored | **0.21** | 0.08, 0.35 | 0.07 | .002 | .13 |
| Feeling disfavored | **0.14** | 0.00, 0.28 | 0.07 | .047 | .08 |
| **PACNR partner effects** |  |  |  |  |  |
| Competition | 0.07 | -0.05, 0.19 | 0.06 | .242 | .04 |
| Feeling favored | 0.09 | -0.04, 0.23 | 0.07 | .188 | .05 |
| Feeling disfavored | 0.13 | -0.01, 0.27 | 0.07 | .065 | .08 |
| **PACPR actor effects** |  |  |  |  |  |
| Competition | 0.08 | -0.02, 0.19 | 0.05 | .127 | .05 |
| Feeling favored | **0.14** | 0.02, 0.25 | 0.06 | .017 | .08 |
| Feeling disfavored | 0.09 | -0.02, 0.20 | 0.06 | .105 | .05 |
| **PACPR partner effects** |  |  |  |  |  |
| Competition | **0.07** | -0.03, 0.21 | 0.05 | .165 | .04 |
| Feeling favored | 0.09 | -0.03, 0.20 | 0.06 | .139 | .05 |
| Feeling disfavored | 0.11 | -0.00, 0.23 | 0.06 | .052 | .07 |
| **PECNR actor effects** |  |  |  |  |  |
| Competition | 0.09 | -0.02, 0.20 | 0.06 | .104 | .05 |
| Feeling favored | **0.14** | 0.03, 0.25 | 0.06 | .013 | .08 |
| Feeling disfavored | 0.09 | -0.02, 0.21 | 0.06 | .102 | .05 |
| **PECNR partner effects** |  |  |  |  |  |
| Competition | 0.05 | -0.05, 0.15 | 0.05 | .340 | .03 |
| Feeling favored | 0.07 | -0.03, 0.17 | 0.05 | .146 | .04 |
| Feeling disfavored | 0.10 | -0.01, 0.20 | 0.05 | .060 | .06 |
| **PECPR actor effects** |  |  |  |  |  |
| Competition | 0.04 | -0.06, 0.14 | 0.05 | .405 | .03 |
| Feeling favored | 0.06 | -0.04, 0.17 | 0.05 | .241 | .04 |
| Feeling disfavored | 0.06 | -0.05, 0.16 | 0.05 | .275 | .03 |
| **PECPR partner effects** |  |  |  |  |  |
| Competition | 0.08 | -0.02, 0.17 | 0.05 | .136 | .04 |
| Feeling favored | **0.12** | -0.02, 0.22 | 0.05 | .021 | .07 |
| Feeling disfavored | **0.11** | 0.02, 0.21 | 0.05 | .023 | .07 |

*Note. N* = 201 sibling dyads. Unstandardized Regression Coefficients (b), Bootstrapped 95%-Confidence Intervals (CIs), Standard Errors (SE), and Standardized Regression Coefficients (β).

O = older siblings, Y = younger siblings.

Bold coefficients indicate that bootstrapped (*k* = 5,000) 95% CIs do not contain zero.

PACPR = parental academic conditional positive regard; PACNR = parental academic conditional negative regard; PECPR = parental emotional conditional positive regard; PECNR = parental emotional conditional negative regard.

Analyses are controlled for the gender of both siblings, maternal educational level and the age and grades of the younger sibling.

Table S4. Indirect actor-actor effects for simple APIMeMs predicting sibling conflict by parental conditional regard, mediated by competition and feeling favored/disfavored.

|  | *b*_O/Y_ | 95%-CI_O/Y_ | *SE*_O/Y_ | *p*_O/Y_ |
| --- | --- | --- | --- | --- |
| Actor-Actor (*b*=aA*bA) |  |  |  |  |
| **PACNR** |  |  |  |  |
| Competition | **0.10** | 0.05, 0.17 | .03 | .000 |
| Actor-Partner | **0.02** | 0.05, 0.17 | .01 | .039 |
| Feeling favored | 0.03 | -0.00, 0.06 | .02 | .112 |
| Feeling disfavored | **0.10** | 0.04, 0.16 | .03 | .003 |
| **PACPR** |  |  |  |  |
| Competition | **0.08** | 0.04, 0.12 | .02 | .000 |
| Actor-Partner | **0.02** | 0.00, 0.05 | .01 | .026 |
| Feeling favored | 0.03 | -0.00, 0.05 | .01 | .085 |
| Feeling disfavored | **0.10/0.04** | 0.04, 0.17/0.01, 0.08 | .03/.02 | .002/.018 |
| **PECNR** |  |  |  |  |
| Competition | **0.06** | 0.03, 0.11 | .02 | .002 |
| Feeling favored | 0.02 | 0.00, 0.04 | .01 | .071 |
| Feeling disfavored | **0.06** | 0.03, 0.11 | .02 | .002 |
| **PECPR** |  |  |  |  |
| Competition | 0.03 | -0.01, 0.08 | .02 | .110 |
| Partner-Actor | 0.04 | 0.00, 0.08 | .02 | .052 |
| Feeling favored | **0.03** | 0.00, 0.04 | .01 | .040 |
| Feeling disfavored | **0.03** | 0.01, 0.06 | .01 | .012 |

*Note.* Unstandardized indirect Actor-Actor-Effects (*b*=aA*bP); Actor-Partner-Effects (*b*=aA*bP) are displayed if significant. Bootstrapped 95%-Confidence Intervals (CIs), Standard Errors (*SE*).

O = older siblings, Y = younger siblings.

PACPR = parental academic conditional positive regard; PACNR = parental academic conditional positive regard; PECPR = parental emotional conditional positive regard; PECNR = parental emotional conditional negative regard.

Analyses are controlled for the gender of both siblings, maternal educational level and the age and grades of the younger sibling.

Table S5. Overall APIMeM predicting sibling conflict by parental academic conditional regard, mediated by competition, feeling favored and feeling disfavored.

| **a-paths**  **Parenting 🡪 Mediator** | ***b*_O/Y_** | **95%-CI_O/Y_** | ***SE*_O/Y_** | ***p*_O/Y_** | **β_O/Y_** |
| --- | --- | --- | --- | --- | --- |
| **PACNR actor effects** |  |  |  |  |  |
| Competition | 0.04/  **0.26** | -0.21, 0.30/  -0.02, 0.50 | 0.13/  0.13 | .741/  .042 | .02/  **.14** |
| Feeling favored | 0.12 | -0.06, 0.30 | 0.09 | .199 | .08 |
| Feeling disfavored | **0.42** | 0.20, 0.62 | 0.11 | .000 | **.26** |
| **PACNR partner effects** |  |  |  |  |  |
| Competition | 0.01 | -0.18, 0.23 | 0.10 | .919 | .01 |
| Feeling favored | 0.08 | -0.09, 0.25 | 0.09 | .333 | .06 |
| Feeling disfavored | -0.09 | -0.28, 0.09 | 0.10 | .349 | -.06 |
| **PACPR actor effects** |  |  |  |  |  |
| Competition | **0.18** | 0.03, 0.34 | 0.08 | .025 | **.10** |
| Feeling favored | **0.17** | 0.03, 0.31 | 0.07 | .017 | **.11** |
| Feeling disfavored | **0.33**/  0.04 | 0.12, 0.52/  -0.13, 0.21 | 0.10/  0.09 | .001/  .679 | **.19**/  .03 |
| **PACPR partner effects** |  |  |  |  |  |
| Competition | -0.02 | -0.17, 0.13 | 0.08 | .826 | -.01 |
| Feeling favored | 0.03 | -0.09, 0.16 | 0.06 | .630 | .02 |
| Feeling disfavored | 0.01 | -0.16, 0.19 | 0.09 | .885 | .01 |
| **b-paths**  **Mediator 🡪 Conflict** | ***b*_O/Y_** |  |  |  |  |
| **actor effects** |  |  |  |  |  |
| Competition | **0.30** | 0.22, 0.39 | 0.04 | .000 | **.18** |
| Feeling favored | 0.01 | -0.12, 0.13 | 0.07 | .917 | .00 |
| Feeling disfavored | **0.11** | 0.00, 0.23 | 0.06 | .049 | **.07** |
| **partner effects** |  |  |  |  |  |
| Competition | **0.10** | 0.02, 0.18 | 0.04 | .011 | **.06** |
| Feeling favored | -0.03 | -0.15, 0.09 | 0.06 | .627 | -.02 |
| Feeling disfavored | -0.03 | -0.15, 0.09 | 0.06 | .603 | -.02 |
| **c-paths**  **Parenting 🡪 Conflict** | ***b*_O/Y_** |  |  |  |  |
| **actor effects** |  |  |  |  |  |
| PACNR | 0.08 | -0.10, 0.27 | 0.09 | .368 | .05 |
| PACPR | 0.00 | -0.15, 0.14 | 0.07 | .976 | .00 |
| **partner effects** |  |  |  |  |  |
| PACNR | 0.06 | -0.11, 0.23 | 0.09 | .479 | .04 |
| PACPR | 0.06 | -0.09, 0.20 | 0.07 | .435 | .03 |

*Note. N* = 201 sibling dyads. Unstandardized Regression Coefficients (b), Bootstrapped 95%-Confidence Intervals (CIs), Standard Errors (SE), and Standardized Regression Coefficients (β).

O = older siblings, Y = younger siblings.

Bold coefficients indicate that bootstrapped (*k* = 5,000) 95% CIs do not contain zero.

PACPR = parental academic conditional positive regard; PACNR = parental academic conditional negative regard.

Analyses are controlled for the gender of both siblings, maternal educational level and the age and grades of the younger sibling.

Table S6. Overall APIMeM predicting sibling conflict by parental emotional conditional regard, mediated by competition, feeling favored and feeling disfavored.

| **a-paths**  **Parenting 🡪 Mediator** | ***b*_O/Y_** | **95%-CI_O/Y_** | ***SE*_O/Y_** | ***p*_O/Y_** | **β_O/Y_** |
| --- | --- | --- | --- | --- | --- |
| **PECNR actor effects** |  |  |  |  |  |
| Competition | **0.17** | 0.04, 0.28 | 0.06 | .009 | **.09** |
| Feeling favored | 0.09 | -0.01, 0.2 | 0.05 | .096 | .06 |
| Feeling disfavored | **0.30** | 0.17, 0.42 | 0.06 | .000 | **.19** |
| **PECNR partner effects** |  |  |  |  |  |
| Competition | 0.02 | -0.1, 0.14 | 0.06 | .727 | .01 |
| Feeling favored | 0.04 | -0.06, 0.14 | 0.05 | .414 | .03 |
| Feeling disfavored | -0.04 | -0.14, 0.04 | 0.05 | .348 | -.03 |
| **PECPR actor effects** |  |  |  |  |  |
| Competition | 0.03 | -0.1, 0.16 | 0.07 | .621 | .02 |
| Feeling favored | 0.16 | 0.06, 0.27 | 0.05 | .002 | **.11** |
| Feeling disfavored | 0.06 | -0.05, 0.17 | 0.06 | .328 | .04 |
| **PECPR partner effects** |  |  |  |  |  |
| Competition | 0.09 | -0.02, 0.21 | 0.06 | .131 | .05 |
| Feeling favored | -0.03 | -0.13, 0.07 | 0.05 | .582 | -.02 |
| Feeling disfavored | 0.04 | -0.06, 0.15 | 0.05 | .414 | .03 |
| **b-paths**  **Mediator 🡪 Conflict** |  |  |  |  |  |
| **actor effects** |  |  |  |  |  |
| Competition | 0.30 | 0.21, 0.39 | 0.05 | .000 | **.17** |
| Feeling favored | 0.02 | -0.12, 0.14 | 0.07 | .806 | .01 |
| Feeling disfavored | 0.12 | 0.01, 0.23 | 0.06 | .038 | **.07** |
| **partner effects** |  |  |  |  |  |
| Competition | 0.10 | 0.02, 0.19 | 0.04 | .015 | **.06** |
| Feeling favored | -0.03 | -0.15, 0.09 | 0.06 | .630 | -.02 |
| Feeling disfavored | -0.02 | -0.14, 0.1 | 0.06 | .742 | -.01 |
| **c-paths**  **Parenting 🡪 Conflict** |  |  |  |  |  |
| **actor effects** |  |  |  |  |  |
| PACNR | 0.04 | -0.08, 0.16 | 0.06 | .503 | .02 |
| PACPR | 0.00 | -0.11, 0.11 | 0.05 | .941 | .00 |
| **partner effects** |  |  |  |  |  |
| PACNR | 0.04 | -0.08, 0.15 | 0.06 | .459 | .02 |
| PACPR | 0.06 | -0.04, 0.17 | 0.05 | .254 | .04 |

*Note. N* = 201 sibling dyads. Unstandardized Regression Coefficients (b), Bootstrapped 95%-Confidence Intervals (CIs), Standard Errors (SE), and Standardized Regression Coefficients (β).

O = older siblings, Y = younger siblings.

Bold coefficients indicate that bootstrapped (*k* = 5,000) 95% CIs do not contain zero.

PACPR = parental academic conditional positive regard; PACNR = parental academic conditional negative regard.

Analyses are controlled for the gender of both siblings, maternal educational level and the age and grades of the younger sibling.

Table S7. Control variables for the overall academic and emotional APIMeM

| **Academic model** |  | ***b*** | **95%-CI** | ***SE*** | ***p*** | **β** |
| --- | --- | --- | --- | --- | --- | --- |
| Competition (O) | g (O) | -0.77 | -1.27, -0.29 | 0.25 | .002 | **-.42** |
|  | g (Y) | -0.638 | -1.13, -0.13 | 0.26 | .014 | **-.35** |
|  | SES | 0.111 | -0.04, 0.26 | 0.07 | .133 | .06 |
|  | age (Y) | -0.189 | -0.37, -0.01 | 0.09 | .036 | -.10 |
|  | grade (Y) | 0.017 | -0.1, 0.15 | 0.06 | .792 | .01 |
| Competition (Y) | g (O) | -0.443 | -0.97, 0.07 | 0.27 | .098 | -.24 |
|  | g (Y) | -0.542 | -1.07, -0.01 | 0.27 | .046 | **-.29** |
|  | SES | 0.122 | -0.02, 0.26 | 0.07 | .101 | .07 |
|  | age (Y) | -0.144 | -0.31, 0.01 | 0.08 | .069 | -.08 |
|  | grade (Y) | -0.007 | -0.13, 0.12 | 0.06 | .907 | .00 |
| Feeling favored (O) | g (O) | -0.381 | -0.75, -0.03 | 0.19 | .040 | **-.28** |
|  | g (Y) | 0.291 | -0.06, 0.65 | 0.18 | .112 | .21 |
|  | SES | 0.067 | -0.04, 0.17 | 0.05 | .197 | .05 |
|  | age (Y) | 0.023 | -0.09, 0.13 | 0.06 | .684 | .02 |
|  | grade (Y) | 0.046 | -0.04, 0.14 | 0.04 | .282 | .03 |
| Feeling favored (Y) | g (O) | 0.089 | -0.37, 0.54 | 0.23 | .701 | .06 |
|  | g (Y) | -0.58 | -1.04, -0.12 | 0.23 | .012 | **-.36** |
|  | SES | -0.033 | -0.17, 0.1 | 0.07 | .633 | -.02 |
|  | age (Y) | -0.014 | -0.15, 0.13 | 0.07 | .844 | -.01 |
|  | grade (Y) | 0.043 | -0.08, 0.16 | 0.06 | .500 | .03 |
| Feeling disfavored (O) | g (O) | 0.323 | -0.13, 0.74 | 0.22 | .146 | .18 |
|  | g (Y) | 0.223 | -0.22, 0.67 | 0.23 | .323 | .13 |
|  | SES | 0.155 | 0.02, 0.28 | 0.07 | .019 | **.09** |
|  | age (Y) | -0.025 | -0.17, 0.12 | 0.07 | .728 | -.01 |
|  | grade (Y) | 0.085 | -0.02, 0.18 | 0.05 | .092 | .05 |
| Feeling disfavored (Y) | g (O) | 0.066 | -0.33, 0.45 | 0.20 | .739 | .05 |
|  | g (Y) | -0.061 | -0.46, 0.34 | 0.20 | .763 | -.04 |
|  | SES | 0.035 | -0.08, 0.15 | 0.06 | .536 | .02 |
|  | age (Y) | 0.122 | 0.01, 0.25 | 0.06 | .043 | **.09** |
|  | grade (Y) | -0.016 | -0.1, 0.07 | 0.04 | .720 | -.01 |
| Conflict (O) | g (O) | -0.105 | -0.54, 0.34 | 0.23 | .642 | -.06 |
|  | g (Y) | 0.175 | -0.26, 0.6 | 0.21 | .413 | .10 |
|  | SES | -0.174 | -0.3, -0.06 | 0.06 | .006 | **-.10** |
|  | age (Y) | -0.054 | -0.17, 0.06 | 0.06 | .382 | -.03 |
|  | grade (Y) | 0.079 | -0.03, 0.18 | 0.05 | .141 | .05 |
| Conflict (Y) | g (O) | 0.239 | -0.23, 0.68 | 0.23 | .301 | .14 |
|  | g (Y) | -0.003 | -0.43, 0.42 | 0.22 | .989 | .00 |
|  | SES | -0.279 | -0.4, -0.15 | 0.06 | .000 | **-.16** |
|  | age (Y) | -0.22 | -0.35, -0.11 | 0.06 | .000 | **-.13** |
|  | grade (Y) | 0.119 | 0.01, 0.22 | 0.05 | .023 | **.07** |
| **Emotional Model** |  | ***b*** | **95%-CI** | ***SE*** | ***p*** | **β** |
| Competition (O) | g (O) | -0.74 | -1.26, -0.24 | 0.26 | .004 | **-.44** |
|  | g (Y) | -0.58 | -1.08, -0.07 | 0.26 | .025 | **-.35** |
|  | SES | 0.05 | -0.10, 0.20 | 0.08 | .471 | .03 |
|  | age (Y) | -0.18 | -0.36, 0.00 | 0.09 | .055 | -.11 |
|  | grade (Y) | 0.03 | -0.09, 0.16 | 0.06 | .614 | .02 |
| Competition (Y) | g (O) | -0.50 | -0.99, 0.00 | 0.26 | .053 | -.29 |
|  | g (Y) | -0.67 | -1.19, -0.15 | 0.27 | .011 | **-.39** |
|  | SES | 0.10 | -0.04, 0.24 | 0.07 | .167 | .06 |
|  | age (Y) | -0.16 | -0.33, -0.01 | 0.08 | .048 | **-.09** |
|  | grade (Y) | 0.02 | -0.11, 0.15 | 0.07 | .825 | .01 |
| Feeling favored (O) | g (O) | -0.39 | -0.76, -0.03 | 0.19 | .038 | **-.28** |
|  | g (Y) | 0.24 | -0.12, 0.61 | 0.19 | .200 | .17 |
|  | SES | 0.03 | -0.07, 0.13 | 0.05 | .532 | .02 |
|  | age (Y) | 0.03 | -0.08, 0.14 | 0.06 | .569 | .02 |
|  | grade (Y) | 0.05 | -0.04, 0.15 | 0.05 | .276 | .04 |
| Feeling favored (Y) | g (O) | 0.01 | -0.43, 0.46 | 0.23 | .956 | .01 |
|  | g (Y) | -0.59 | -1.04, -0.13 | 0.23 | .009 | **-.37** |
|  | SES | -0.06 | -0.20, 0.07 | 0.07 | .348 | -.04 |
|  | age (Y) | -0.04 | -0.18, 0.11 | 0.07 | .582 | -.02 |
|  | grade (Y) | 0.08 | -0.04, 0.20 | 0.06 | .200 | .05 |
| Feeling disfavored (O) | g (O) | 0.26 | -0.19, 0.70 | 0.23 | .255 | .15 |
|  | g (Y) | 0.23 | -0.23, 0.70 | 0.23 | .320 | .13 |
|  | SES | 0.06 | -0.07, 0.18 | 0.06 | .352 | .03 |
|  | age (Y) | -0.01 | -0.17, 0.18 | 0.09 | .920 | -.01 |
|  | grade (Y) | 0.11 | -0.01, 0.21 | 0.06 | .065 | .06 |
| Feeling disfavored (Y) | g (O) | 0.03 | -0.38, 0.44 | 0.21 | .891 | .02 |
|  | g (Y) | -0.11 | -0.51, 0.31 | 0.21 | .595 | -.08 |
|  | SES | 0.03 | -0.09, 0.14 | 0.06 | .620 | .02 |
|  | age (Y) | 0.10 | -0.03, 0.22 | 0.06 | .115 | .07 |
|  | grade (Y) | 0.01 | -0.08, 0.11 | 0.05 | .781 | .01 |
| Conflict (O) | g (O) | -0.15 | -0.58, 0.29 | 0.22 | .512 | -.08 |
|  | g (Y) | 0.15 | -0.27, 0.57 | 0.22 | .485 | .08 |
|  | SES | -0.18 | -0.32, -0.06 | 0.07 | .005 | **-.10** |
|  | age (Y) | -0.07 | -0.18, 0.06 | 0.06 | .281 | -.04 |
|  | grade (Y) | 0.09 | -0.01, 0.20 | 0.05 | .076 | .05 |
| Conflict (Y) | g (O) | 0.22 | -0.24, 0.66 | 0.23 | .338 | .12 |
|  | g (Y) | -0.02 | -0.47, 0.41 | 0.23 | .917 | -.01 |
|  | SES | -0.29 | -0.41, -0.16 | 0.07 | .000 | **-.16** |
|  | age (Y) | -0.22 | -0.35, -0.11 | 0.06 | .000 | **-.12** |
|  | grade (Y) | 0.12 | 0.02, 0.22 | 0.05 | .016 | **.07** |

*Note. N* = 201 sibling dyads. Unstandardized Regression Coefficients (b), Bootstrapped 95%-Confidence Intervals (CIs), Standard Errors (SE), and Standardized Regression Coefficients (β).

O = older siblings, Y = younger siblings.

Bold coefficients indicate that bootstrapped (*k* = 5,000) 95% CIs do not contain zero.

Analyses are controlled for the gender of both siblings, maternal educational level and the age and grades of the younger sibling.

Table S8. Fit indices of restricted simple APIMeM without control variables predicting sibling conflict by parental conditional regard, mediated by competition and feeling favored/disfavored.

|  | χ² | *df* | *p* | *CFI* | *RMSEA* | *SRMR* |
| --- | --- | --- | --- | --- | --- | --- |
| **PACNR** |  |  |  |  |  |  |
| Competition | 5.879 | 6 | .437 | 1.000 | .000 | .030 |
| Feeling favored | 1.987 | 6 | .921 | 1.000 | .000 | .017 |
| Feeling disfavored | 4.763 | 6 | .575 | 1.000 | .000 | .034 |
| **PACPR** |  |  |  |  |  |  |
| Competition | 7.177 | 6 | .305 | .993 | .031 | .033 |
| Feeling favored | 2.842 | 6 | .828 | 1.000 | .000 | .022 |
| Feeling disfavored | 11.955 | 6 | .063 | .958 | .070 | .051 |
| Feeling disfavored, specific | 4.154 | 5 | .528 | 1.000 | .000 | .027 |
| **PECPR** |  |  |  |  |  |  |
| Competition | 7.312 | 6 | .293 | .992 | .033 | .036 |
| Feeling favored | 0.939 | 6 | .988 | 1.000 | .000 | .013 |
| Feeling disfavored | 6.919 | 6 | .328 | .993 | .028 | .041 |
| **PECNR** |  |  |  |  |  |  |
| Competition | 8.457 | 6 | .201 | .984 | .045 | .038 |
| Feeling favored | 1.879 | 6 | .931 | 1.000 | .000 | .016 |
| Feeling disfavored | 3.382 | 6 | .760 | 1.000 | .000 | .024 |

*Note.* Resricted models are those, in which all actor and partner effects are set equal for older and younger siblings (df = 6). If *p* < .20 for the completely restricted models (bold values), we freed specific paths, to find the most restricted model, which is still not distinct from the fully identified model (df = 5).

PACPR = parental academic conditional positive regard; PACNR = parental academic conditional negative regard; PECPR = parental emotional conditional positive regard; PECNR = parental emotional conditional negative regard.

Table S9. Simple APIMeMs without control variables predicting sibling conflict by parental conditional regard, mediated by competition and feeling favored/disfavored.

| **a-paths**  **Parenting 🡪 Mediator** | ***b*_O/Y_** | **95%-CI_O/Y_** | ***SE*_O/Y_** | ***p*_O/Y_** | **β_O/Y_** |
| --- | --- | --- | --- | --- | --- |
| **PACNR actor effects** |  |  |  |  |  |
| Competition | 0.19 | 0.06, 0.32 | 0.07 | .004 | .10 |
| Feeling favored | 0.27 | 0.14, 0.42 | 0.07 | .000 | .18 |
| Feeling disfavored | 0.53 | 0.37, 0.68 | 0.08 | .000 | .33 |
| **PACNR partner effects** |  |  |  |  |  |
| Competition | 0.09 | -0.04, 0.23 | 0.07 | .164 | .05 |
| Feeling favored | 0.14 | 0.00, 0.28 | 0.07 | .047 | .09 |
| Feeling disfavored | -0.06 | -0.17, 0.06 | 0.06 | .325 | -.04 |
| **PACPR actor effects** |  |  |  |  |  |
| Competition | 0.24 | 0.13, 0.35 | 0.06 | .000 | .13 |
| Feeling favored | 0.26 | 0.15, 0.37 | 0.06 | .000 | .17 |
| Feeling disfavored | 0.51/  0.23 | 0.34, 0.69/  0.08, 0.38 | 0.09/  0.08 | .000/  .003 | .29/  .16 |
| **PACPR partner effects** |  |  |  |  |  |
| Competition | 0.00 | -0.12, 0.13 | 0.06 | .960 | .00 |
| Feeling favored | 0.07 | -0.04, 0.17 | 0.05 | .216 | .04 |
| Feeling disfavored | -0.02 | -0.13, 0.11 | 0.06 | .776 | -.01 |
| **PECNR actor effects** |  |  |  |  |  |
| Competition | 0.22 | 0.10, 0.33 | 0.06 | .000 | .12 |
| Feeling favored | 0.16 | 0.07, 0.25 | 0.05 | .001 | .11 |
| Feeling disfavored | 0.33 | 0.22, 0.44 | 0.06 | .000 | .21 |
| **PECNR partner effects** |  |  |  |  |  |
| Competition | 0.07 | -0.04, 0.19 | 0.06 | .229 | .04 |
| Feeling favored | 0.03 | -0.06, 0.12 | 0.05 | .535 | .02 |
| Feeling disfavored | -0.04 | -0.12, 0.04 | 0.04 | .385 | -.02 |
| **PECPR actor effects** |  |  |  |  |  |
| Competition | 0.13 | 0.00, 0.25 | 0.06 | .043 | .07 |
| Feeling favored | 0.23 | 0.13, 0.32 | 0.05 | .000 | .15 |
| Feeling disfavored | 0.17 | 0.07, 0.27 | 0.05 | .001 | .11 |
| **PECPR partner effects** |  |  |  |  |  |
| Competition | 0.13 | 0.02, 0.25 | 0.06 | .025 | .07 |
| Feeling favored | -0.01 | -0.10, 0.09 | 0.05 | .900 | .00 |
| Feeling disfavored | 0.05 | -0.04, 0.15 | 0.05 | .303 | .03 |
| **b-paths**  **Mediator 🡪 Conflict** |  |  |  |  |  |
| **PACNR actor effects** |  |  |  |  |  |
| Competition | 0.32 | 0.17, 0.46 | 0.07 | .000 | .18 |
| Feeling favored | 0.13 | 0.02, 0.25 | 0.06 | .021 | .08 |
| Feeling disfavored | 0.13 | 0.03, 0.24 | 0.06 | .016 | .08 |
| **PACNR partner effects** |  |  |  |  |  |
| Competition | 0.07 | -0.09, 0.23 | 0.08 | .416 | .04 |
| Feeling favored | 0.02 | -0.10, 0.14 | 0.06 | .721 | .01 |
| Feeling disfavored | -0.04 | -0.16, 0.08 | 0.06 | .477 | -.02 |
| **PACPR actor effects** |  |  |  |  |  |
| Competition | 0.31 | 0.22, 0.40 | 0.05 | .000 | .18 |
| Feeling favored | 0.13 | 0.02, 0.24 | 0.06 | .020 | .08 |
| Feeling disfavored | 0.14 | 0.04, 0.25 | 0.05 | .007 | .08 |
| **PACPR partner effects** |  |  |  |  |  |
| Competition | 0.07 | -0.01, 0.16 | 0.04 | .075 | .04 |
| Feeling favored | 0.01 | -0.10, 0.13 | 0.06 | .838 | .01 |
| Feeling disfavored | -0.05 | -0.16, 0.06 | 0.06 | .377 | -.03 |
| **PECNR actor effects** |  |  |  |  |  |
| Competition | 0.32 | 0.23, 0.41 | 0.05 | .000 | .19 |
| Feeling favored | 0.17 | 0.06, 0.28 | 0.06 | .003 | .10 |
| Feeling disfavored | 0.17 | 0.06, 0.28 | 0.06 | .002 | .10 |
| **PECNR partner effects** |  |  |  |  |  |
| Competition | 0.09 | 0.00, 0.17 | 0.04 | .042 | .05 |
| Feeling favored | 0.05 | -0.06, 0.16 | 0.06 | .334 | .03 |
| Feeling disfavored | -0.02 | -0.13, 0.09 | 0.06 | .763 | -.01 |
| **PECPR actor effects** |  |  |  |  |  |
| Competition | 0.32 | 0.23, 0.41 | 0.05 | .000 | .19 |
| Feeling favored | 0.17 | 0.06, 0.28 | 0.06 | .002 | .10 |
| Feeling disfavored | 0.18 | 0.07, 0.28 | 0.05 | .001 | .10 |
| **PECPR partner effects** |  |  |  |  |  |
| Competition | 0.08 | -0.01, 0.16 | 0.04 | .058 | .05 |
| Feeling favored | 0.03 | -0.08, 0.14 | 0.06 | .596 | .02 |
| Feeling disfavored | -0.02 | -0.13, 0.08 | 0.06 | .682 | -.01 |
| **c-paths**  **Parenting 🡪 Conflict** |  |  |  |  |  |
| **PACNR actor effects** |  |  |  |  |  |
| Competition | 0.31 | 0.22, 0.40 | 0.05 | .000 | .18 |
| Feeling favored | 0.25 | 0.12, 0.39 | 0.07 | .000 | .15 |
| Feeling disfavored | 0.22 | 0.08, 0.36 | 0.07 | .002 | .13 |
| **PACNR partner effects** |  |  |  |  |  |
| Competition | 0.08 | -0.01, 0.16 | 0.04 | .063 | .05 |
| Feeling favored | 0.11 | -0.02, 0.25 | 0.07 | .105 | .07 |
| Feeling disfavored | 0.17 | 0.02, 0.31 | 0.07 | .025 | .10 |
| **PACPR actor effects** |  |  |  |  |  |
| Competition | 0.14 | 0.03, 0.26 | 0.06 | .013 | .08 |
| Feeling favored | 0.18 | 0.07, 0.29 | 0.06 | .002 | .11 |
| Feeling disfavored | 0.16 | 0.05, 0.28 | 0.06 | .006 | .09 |
| **PACPR partner effects** |  |  |  |  |  |
| Competition | 0.12 | 0.01, 0.23 | 0.06 | .036 | .07 |
| Feeling favored | 0.13 | 0.01, 0.25 | 0.06 | .027 | .08 |
| Feeling disfavored | 0.16 | 0.04, 0.28 | 0.06 | .006 | .09 |
| **PECNR actor effects** |  |  |  |  |  |
| Competition | 0.08 | -0.03, 0.19 | 0.06 | .174 | .04 |
| Feeling favored | 0.12 | 0.01, 0.22 | 0.06 | .031 | .07 |
| Feeling disfavored | 0.09 | -0.02, 0.20 | 0.06 | .124 | .05 |
| **PECNR partner effects** |  |  |  |  |  |
| Competition | 0.02 | -0.09, 0.12 | 0.05 | .765 | .01 |
| Feeling favored | 0.04 | -0.06, 0.14 | 0.05 | .434 | .02 |
| Feeling disfavored | 0.07 | -0.04, 0.18 | 0.06 | .217 | .04 |
| **PECPR actor effects** |  |  |  |  |  |
| Competition | 0.05 | -0.06, 0.15 | 0.05 | .349 | .03 |
| Feeling favored | 0.06 | -0.05, 0.17 | 0.05 | .262 | .04 |
| Feeling disfavored | 0.07 | -0.03, 0.18 | 0.05 | .187 | .04 |
| **PECPR partner effects** |  |  |  |  |  |
| Competition | 0.08 | -0.02, 0.19 | 0.05 | .105 | .05 |
| Feeling favored | 0.13 | 0.03, 0.23 | 0.05 | .011 | .08 |
| Feeling disfavored | 0.13 | 0.03, 0.23 | 0.05 | .009 | .08 |

*Note. N* = 201 sibling dyads. Unstandardized Regression Coefficients (b), Bootstrapped 95%-Confidence Intervals (CIs), Standard Errors (SE), and Standardized Regression Coefficients (β).

O = older siblings, Y = younger siblings.

Bold coefficients indicate that bootstrapped (*k* = 5,000) 95% CIs do not contain zero.

PACPR = parental academic conditional positive regard; PACNR = parental academic conditional negative regard; PECPR = parental emotional conditional positive regard; PECNR = parental emotional conditional negative regard.

Table S10. Overall APIMeM without control variables predicting sibling conflict by parental academic conditional regard, mediated by competition, feeling favored and feeling disfavored.

| **a-paths**  **Parenting 🡪 Mediator** | ***b*_O/Y_** | **95%-CI_O/Y_** | ***SE*_O/Y_** | ***p*_O/Y_** | **β_O/Y_** |
| --- | --- | --- | --- | --- | --- |
| **PACNR actor effects** |  |  |  |  |  |
| Competition | 0.24 | 0.01, 0.45 | 0.11 | .037 | **.13** |
| Feeling favored | 0.13 | -0.04, 0.32 | 0.09 | .149 | .09 |
| Feeling disfavored | 0.41 | 0.18, 0.62 | 0.11 | .000 | **.26** |
| **PACNR partner effects** |  |  |  |  |  |
| Competition | 0.09 | -0.10, 0.31 | 0.11 | .395 | .05 |
| Feeling favored | 0.09 | -0.09, 0.26 | 0.09 | .307 | .06 |
| Feeling disfavored | -0.09 | -0.28, 0.10 | 0.10 | .374 | -.05 |
| **PACPR actor effects** |  |  |  |  |  |
| Competition | 0.12 | -0.04, 0.28 | 0.08 | .146 | .06 |
| Feeling favored | 0.18 | 0.04, 0.32 | 0.07 | .015 | **.12** |
| Feeling disfavored | 0.32/  0.02 | 0.13, 0.53/  -0.16, 0.20 | 0.10/  0.09 | .001/  .834 | **.18**/  .01 |
| **PACPR partner effects** |  |  |  |  |  |
| Competition | -0.05 | -0.19, 0.08 | 0.07 | .470 | -.03 |
| Feeling favored | 0.02 | -0.10, 0.15 | 0.06 | .733 | .01 |
| Feeling disfavored | 0.00 | -0.17, 0.18 | 0.09 | .996 | .00 |
| **b-paths**  **Mediator 🡪 Conflict** |  |  |  |  |  |
| **actor effects** |  |  |  |  |  |
| Competition | 0.29 | 0.20, 0.39 | 0.05 | .000 | **.17** |
| Feeling favored | 0.06 | -0.07, 0.19 | 0.07 | .372 | .04 |
| Feeling disfavored | 0.06 | -0.06, 0.17 | 0.06 | .312 | .04 |
| **partner effects** |  |  |  |  |  |
| Competition | 0.08 | -0.01, 0.17 | 0.04 | .060 | .05 |
| Feeling favored | -0.03 | -0.16, 0.10 | 0.07 | .662 | -.02 |
| Feeling disfavored | -0.05 | -0.18, 0.07 | 0.06 | .382 | -.03 |
| **c-paths**  **Parenting 🡪 Conflict** |  |  |  |  |  |
| **actor effects** |  |  |  |  |  |
| PACNR | 0.07 | -0.12, 0.26 | 0.10 | .495 | .04 |
| PACPR | 0.07 | -0.08, 0.23 | 0.08 | .345 | .04 |
| **partner effects** |  |  |  |  |  |
| PACNR | 0.01 | -0.17, 0.2 | 0.09 | .891 | .01 |
| PACPR | 0.13 | -0.02, 0.28 | 0.08 | .083 | .08 |

*Note. χ*²(21) = 17.557, *p* = .677; *CFI* = 1.000, *RMSEA* = .000, *SRMR* = .027.

*N* = 201 sibling dyads. Unstandardized Regression Coefficients (b), Bootstrapped 95%-Confidence Intervals (CIs), Standard Errors (SE), and Standardized Regression Coefficients (β).

O = older siblings, Y = younger siblings.

Bold coefficients indicate that bootstrapped (*k* = 5,000) 95% CIs do not contain zero.

PACPR = parental academic conditional positive regard; PACNR = parental academic conditional negative regard.

Table S11. Overall APIMeM without control variables predicting sibling conflict by parental emotional conditional regard, mediated by competition, feeling favored and feeling disfavored.

| **a-paths**  **Parenting 🡪 Mediator** | ***b*_O/Y_** | **95%-CI_O/Y_** | ***SE*_O/Y_** | ***p*_O/Y_** | **β_O/Y_** |
| --- | --- | --- | --- | --- | --- |
| **PECNR actor effects** |  |  |  |  |  |
| Competition | 0.18 | 0.05, 0.30 | 0.06 | .006 | **.10** |
| Feeling favored | 0.09 | -0.01, 0.20 | 0.05 | .087 | .06 |
| Feeling disfavored | 0.30 | 0.17, 0.42 | 0.06 | .000 | **.19** |
| **PECNR partner effects** |  |  |  |  |  |
| Competition | 0.04 | -0.08, 0.16 | 0.06 | .518 | .02 |
| Feeling favored | 0.02 | -0.09, 0.11 | 0.05 | .772 | .01 |
| Feeling disfavored | -0.05 | -0.15, 0.04 | 0.05 | .303 | -.03 |
| **PECPR actor effects** |  |  |  |  |  |
| Competition | 0.05 | -0.08, 0.19 | 0.07 | .422 | .03 |
| Feeling favored | 0.18 | 0.08, 0.29 | 0.05 | .001 | **.12** |
| Feeling disfavored | 0.07 | -0.04, 0.18 | 0.06 | .246 | .04 |
| **PECPR partner effects** |  |  |  |  |  |
| Competition | 0.10 | -0.02, 0.22 | 0.06 | .125 | .05 |
| Feeling favored | -0.02 | -0.12, 0.08 | 0.05 | .697 | -.01 |
| Feeling disfavored | 0.03 | -0.07, 0.14 | 0.05 | .574 | .02 |
| **b-paths**  **Mediator 🡪 Conflict** |  |  |  |  |  |
| **actor effects** |  |  |  |  |  |
| Competition | 0.30 | 0.20, 0.40 | 0.05 | .000 | **.17** |
| Feeling favored | 0.08 | -0.05, 0.22 | 0.07 | .234 | .05 |
| Feeling disfavored | 0.08 | -0.04, 0.20 | 0.06 | .171 | .05 |
| **partner effects** |  |  |  |  |  |
| Competition | 0.09 | 0.00, 0.18 | 0.05 | .049 | **.05** |
| Feeling favored | -0.02 | -0.16, 0.11 | 0.07 | .779 | -.01 |
| Feeling disfavored | -0.03 | -0.16, 0.10 | 0.06 | .594 | -.02 |
| **c-paths**  **Parenting 🡪 Conflict** |  |  |  |  |  |
| **actor effects** |  |  |  |  |  |
| PACNR | 0.02 | -0.10, 0.14 | 0.06 | .709 | .01 |
| PACPR | 0.01 | -0.10, 0.13 | 0.06 | .846 | .01 |
| **partner effects** |  |  |  |  |  |
| PACNR | 0.01 | -0.12, 0.12 | 0.06 | .940 | .00 |
| PACPR | 0.09 | -0.02, 0.20 | 0.06 | .126 | .05 |

*Note. χ*²(22) = 22.994, *p* = .402; *CFI* = 0.997, *RMSEA* = .015, *SRMR* = .036

*N* = 201 sibling dyads. Unstandardized Regression Coefficients (b), Bootstrapped 95%-Confidence Intervals (CIs), Standard Errors (SE), and Standardized Regression Coefficients (β).

O = older siblings, Y = younger siblings.

Bold coefficients indicate that bootstrapped (*k* = 5,000) 95% CIs do not contain zero.

PACPR = parental academic conditional positive regard; PACNR = parental academic conditional negative regard.

Figure S1. Simple Actor Partner Interdependence Mediation Model


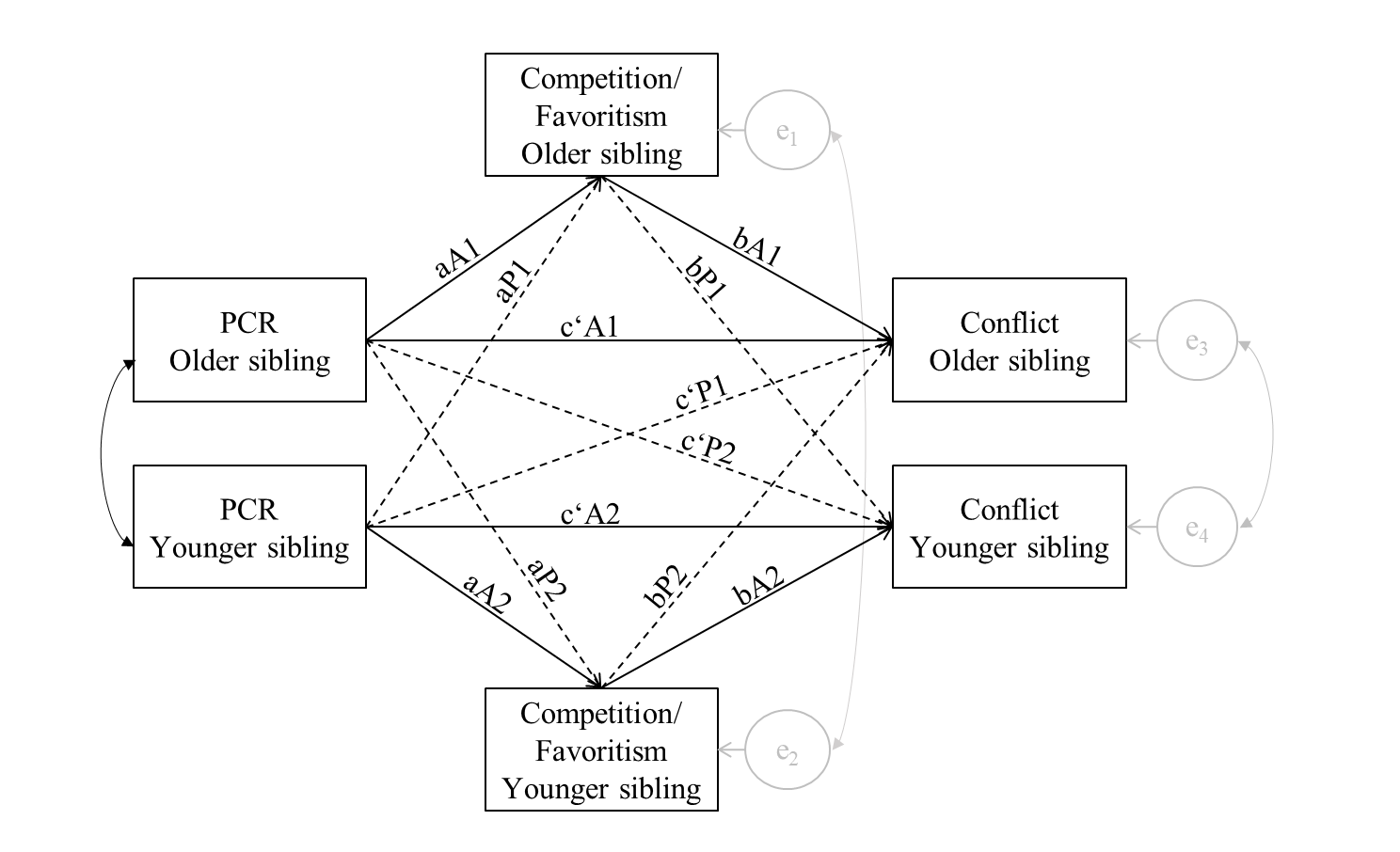


*Note.* a = path from parenting to competition/favoritism; b = path from competition/favoritism to SRQ; c’ = path from parenting to SRQ; A = actor effect; P = partner effect; 1 = effect on older sibling variables; 2 = effect on younger sibling variables.
